# Cu-catalyzed cyanomethylation of imines and α,β-alkenes with acetonitrile and its derivatives†

**DOI:** 10.1039/d0ra10693c

**Published:** 2021-01-28

**Authors:** Muhammad Siddique Ahmad, Atique Ahmad

**Affiliations:** Institute of Chemical Sciences, Bahauddin Zakariya Unversity Multan 66000 Pakistan doctormsahmad@gmail.com; Department of Physical Sciences, Air University, Islamabad Campus Pakistan

## Abstract

We describe copper-catalyzed cyanomethylation of imines and α,β-alkenes with a methylnitrile source and provide an efficient route to synthesize arylacrylonitriles and β,γ-unsaturated nitriles. This method tolerates aliphatic and aromatic alkenes substituted with a variety of functional groups such as F, Cl, Br, Me, OMe, *tert*-Bu, NO_2_, NH_2_ and CO_2_H with good to excellent yields (69–98%). These systems consist of inexpensive, simple copper catalyst and acetonitrile with its derivatives (α-bromo/α-iodo-acetonitrile) and are highly applicable in the industrial production of acrylonitriles.

## Abstract 2

We describe copper-catalyzed cyanomethylation of imines and α,β-alkenes with a methylnitrile source and provide an efficient route to synthesize arylacrylonitriles and β,γ-unsaturated nitriles.

## Graphical abstract

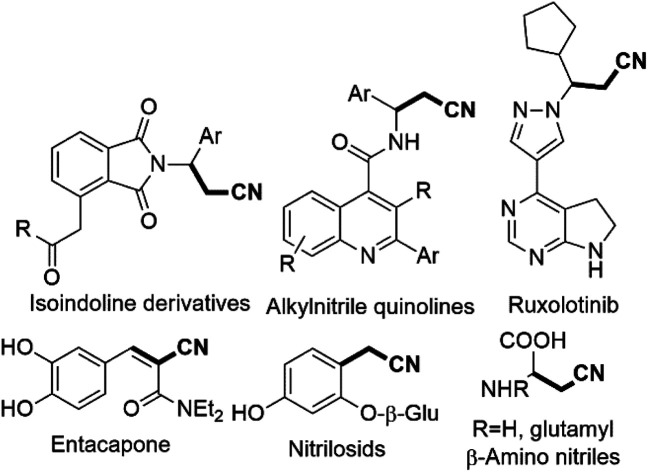

## Introduction and importance

**Scheme 1:**
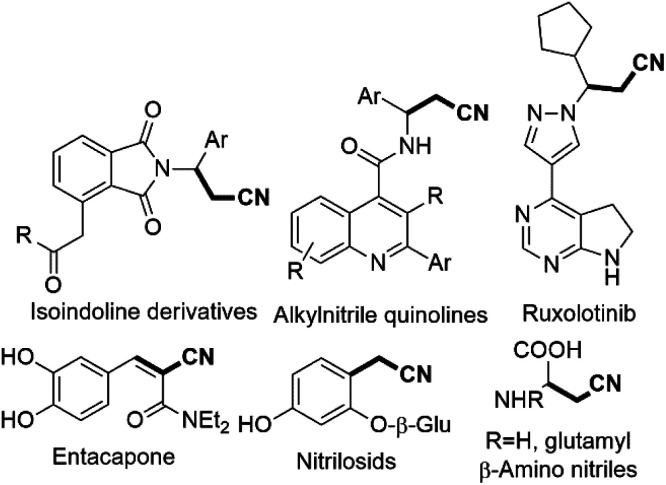
The natural with biologically active alkylnitriles and acrylonitriles.

Acrylonitrile and cyanomethyl are versatile functional units found in many dyes, herbicides, agrochemicals, pharmaceuticals, and natural products.^1^ For example, β,γ-unsaturated nitriles are found in natural products such as alkanenitriles, β-amino nitriles, nitrilosids (Scheme 1).^2^ The biologically active ruxolitinib, alkylnitrile and acrylonitrile containing entacapone are also shown in Scheme 1.^2^ These β,γ-unsaturated nitriles and alkenyl nitriles are also key structural units as antifungal agents and vitamin D receptor.^3^ Besides, the cyano group serves as a valuable intermediate for transformation into aldehydes, amines, amides, tetrazoles, and carboxyl derivatives.^2^ A lot of approaches for the synthesis of β,γ-unsaturated nitriles have been progressed in recent decades.^4^ However, the cyanation of allyl substrates containing leaving groups such as carbonate, or ester alcohol, halide, acetate, phosphate, are frequently used in the transformation into β,γ-unsaturated nitriles.^4*a*–*f*^ Our many efforts have been paid attention in developing non-toxic and slow-releasing cyano-methyl reagents like alkyl nitriles, especially acetonitrile. However, due to its high p*K*_a_ value [p*K*_a_ (CH_3_CN) = 31.3 in DMSO], relatively difficult to be used as a nucleophile. The catalytic C–H bond activation of acetonitrile by transition metals has rarely been explored in last decades.^2*b*^ A few strategies has been reported for cyano-methylation by using acetonitrile for various substrates such as phenazines, 2,2,6,6 tetramethylpipyridine, C_2_-quaternary indolin-3-ones, cycloalkene, simple arenes, aryl-ketone, diarylethenes, azoles, aldehydesaliphatic amides, allylic alcohols, diazonium salts, arylacrylamides, alkenes, 1,3-dicarbonyls, benzaldehyde and coumarins substrates.^5^

**Scheme 2:**
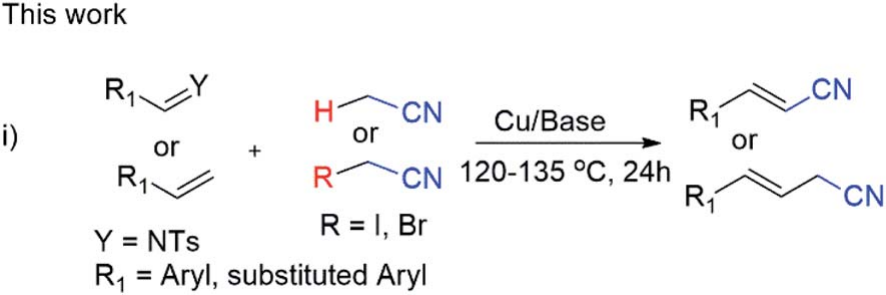
Our Cu-catalyzed cyanomethylation of aromatic imines and styrenes.

Consequently, the reactivity of imines have been rarely explored for chiral cyanomethyl product by transfer of hydrogen atom.^6^ However, synthesis of phenylacrylonitriles from imines not yet explored so for (Scheme 2).^7^ A number of pharmaceutical reagents contain α,β-unsaturated cyanide moiety such as entacapone and rilpvirine, which can be used as anti-Parkinson's and anti-HIV agents.^2^

## Results and discussion

For this copper-catalyzed cyanomethylation of aromatic imines with green MeCN solvent, we used (*E*)-*N*-benzylidene-4-methylbenzenesulfonamide (1a) as a model substrate (Table 1). The compound 1a was treated with Cu(OAc)_2_ (20 mol%) and HOAc (1.0 eq.) under N_2_ atmosphere at 135 °C, which gave cyano-methylated product (2a) in 10% yield (Table 1, entry 1). Moreover, the amination occurs and 4-methylbenzenesulfonamide obtained as directing auxiliary (3a) with low yield (11%) and decomposition of remaining substrate into complex mixture (Table 1, entry 1). For further week acid screening such as HCO_2_H and alcohols (*t*-BuOH, *i*-PrOH) were elaborated low to mild yields (10–39%) (Table 1, entries 2–4). However, strong acid (HCl) unable to produce desired product (Table 1, entry 5). To further explore the reaction parameters, a variety of boronsted bases such as KOBu^*t*^, NaOBu^*t*^, LiOBu^*t*^ and Cs_2_CO_3_ were screened. However, these bases are not suitable for reaction and gave the product (2a) in lower to medium yields (25–51%) (Table 1, entries 6–9). Importantly, Cu(OAc)_2_ evaluated 98% yield of phenylacrylonitrile (2a) with more than 99% of directing auxiliary (4-methylbenzenesulfonamide) in the absence of additive and base or acid (Table 1, entry 10). For further Cu catalyst optimization, a wide variety of Cu(ii) catalysts such as Cu(OTf)_2,_ Cu(ClO_4_)_2_, Cu(C_2_H_5_O_2_)_2_, CuCl_2_, and Cu(i) catalysts (CuI, CuBr, CuCl) were screened (Table 1, entries 11–17).

**Table 1:** Optimization of conditions for imine using CH_3_CN.^a^^,^^b^

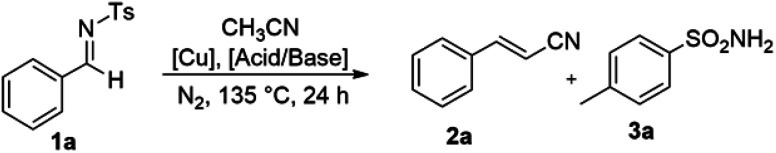					
Entry	[Acid/base]	[Cu(X)_*n*_]	[Cu quant.]	Yield 2a^b^ (%)	Yield 3a^b^ (%)
1	HOAc	Cu(OAc)_2_	20 mol%	10	11
2	HCO_2_H	Cu(OAc)_2_	20 mol%	10	36
3	*t*-BuOH	Cu(OAc)_2_	20 mol%	46	51
4	*i*-PrOH	Cu(OAc)_2_	20 mol%	39	55
5	HCl	Cu(OAc)_2_	20 mol%	0	n.d
6	KO^*t*^Bu	Cu(OAc)_2_	20 mol%	51	60
7	NaO^*t*^Bu	Cu(OAc)_2_	20 mol%	41	53
8	LiO^*t*^Bu	Cu(OAc)_2_	20 mol%	25	40
9	Cs_2_CO_3_	Cu(OAc)_2_	20 mol%	28	49
**10**	**None**	**Cu(OAc)**_**2**_	**20 mol%**	**98**	**>99**
11	None	Cu(OTf)_2_	20 mol%	31	47
12	None	Cu(ClO_4_)_2_	20 mol%	59	71
13	None	Cu(C_2_H_5_O_2_)_2_	20 mol%	68	79
14	None	CuCl_2_	20 mol%	79	81
15	None	CuI	20 mol%	63	88
16	None	CuBr	20 mol%	58	80
17	None	CuCl	20 mol%	46	59
18	None	Cu(OAc)_2_	5 mol%	62	74
19	None	Cu(OAc)_2_	10 mol%	75	83
20	None	Cu(OAc)_2_	15 mol%	88	91
21	None	Cu(OAc)_2_	25 mol%	97	99
22	None	Cu(OAc)_2_	30 mol%	98	99

a Conditions: 1a (0.2 mmol), Cu(X)_*n*_ (Cu-catalysts), acid/base (1.0 eq.), N_2_, 135 °C, CH_3_CN (1.2 mL), 24 h.

b Isolated yield. n.d; not determined.

**Scheme 3:**
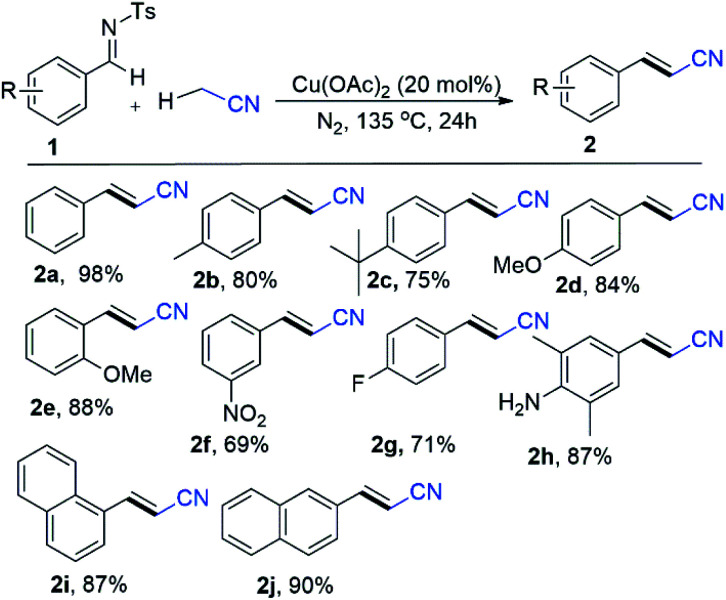
Substrate scope for imines using CH_3_CN.^a,b a^Conditions: 1 (0.2 mmol), Cu(OAc)_2_ (20 mol%), N_2_, 135 °C, CH_3_CN (1.2 mL), 24 h. ^b^Isolated yield.

To our delight, these Cu(ii) catalysts have good reactivity, which gave the corresponding alkenyl cyanated product (2a) in 31% to 79% yield (Table 1, entries 11–14). Moreover, copper(i) halides (I, Br and Cl) catalytic system such as CuI, CuBr and CuCl elaborated cyanomethyl products in 63%, 58% and 46% (Table 1, entries 15–17) respectively. Gratifyingly, all these copper catalysts have worse reactivity than the commercially abundant Cu(OAc)_2_ which produced good yield of phenylacrylonitrile (2a) product. In this context, a various quantities of Cu(OAc)_2_ such as 5 mol%, 10 mol%, 15 mol%, 25 mol% and 30 mol% were examined to find best quantity of Cu(OAc)_2_ as catalyst (Table 1, entries 18–22). Notably, the yields of desired product (2a) dramatically varied when using 5 mol%, 10 mol%, 15 mol%, 25 mol% and 30 mol% of Cu(OAc)_2_ (Table 1, entries 18–22). For example, 62% to 98% yields were obtained by using wide range of quantities for the Cu(OAc)_2_, instead of 20 mol% (Table 1, entries 18–22). After optimization, we elaborate the scope of substrate by varying the substituent on the *N*-benzylidene-4-methylbenzenesulfonamide (Scheme 3). A variety of *N*-benzylidene-4-methylbenzenesulfonamide (1a–1d) with electron-donating group such as Me, OMe, *tert*-butyl at para position of benzene ring afforded the corresponding desired products (2a–2d) in 75% to 98% yields (Scheme 3).

**Scheme 4:**
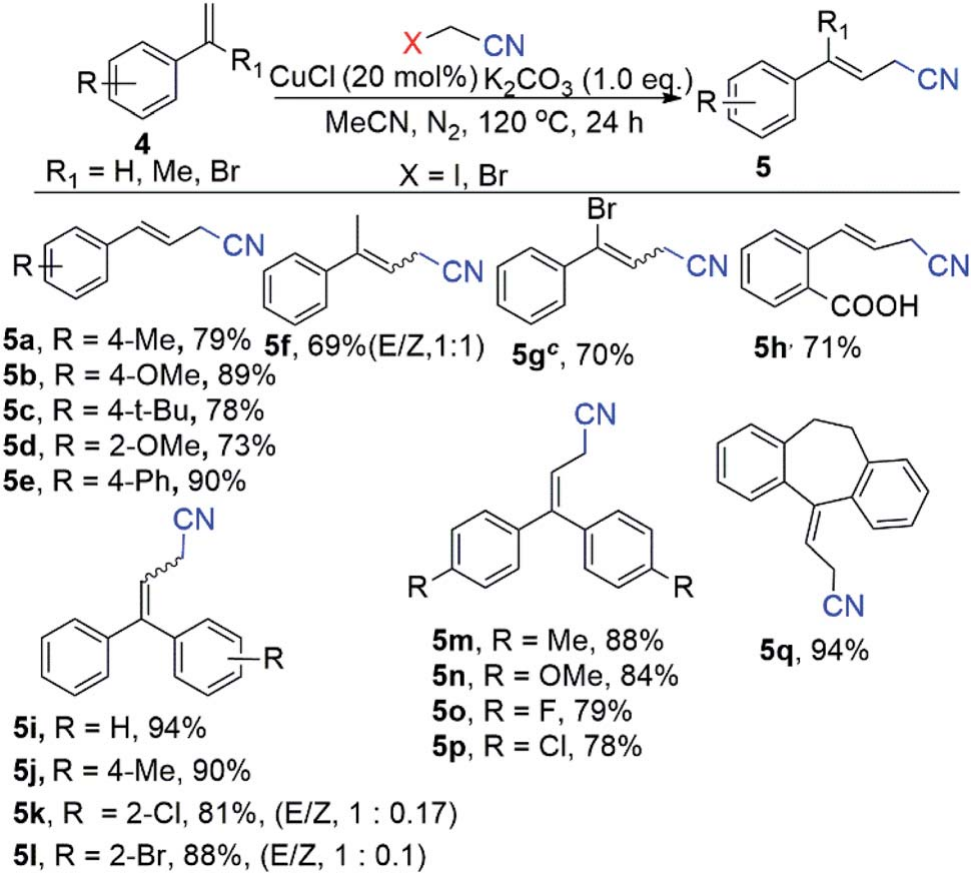
Substrate scope for styrenes using CH_3_CN derivatives.^*a*,*b a*^Conditions: 4 (0.2 mmol), CuCl (20 mol%), K_2_CO_3_ (1.0 eq.), bromoacetonitrile (2.0 eq.), N_2_, 135 °C, CH_3_CN (1.2 mL), 24 h. ^*b*^Isolated yield. ^*c*^iodoacetonitrile (2.0 eq.).

In this context, electron-rich methoxy substituent at ortho position of benzene ring such as (*E*)-*N*-(2-methoxybenzylidene)-4-methylbenzenesulfonamide (1e) efficiently evaluated the corresponding product (2e) in 88% yields (Scheme 3). Gratifyingly, scope of substrate extended to electron sensitive electron functional groups such as nitro (1f), fluoro (1g) and free amino (1h) groups were attached to the benzene of *N*-benzylidene-4-methylbenzenesulfonamide, which worked well and formed aryl-alkenyl cyanated products (1f–1h) in 69%, 71% and 87% yields (Scheme 3) respectively. Delightfully, we used the 4-methyl-*N*-(naphthalen-1-ylmethylene)benzenesulfonamide (1i) and 2-napthalene 4-methyl-*N*-(naphthalen-2-ylmethylene)benzenesulfonamide (1j) for the Cu-catalyzed cyanomethylation and obtained excellent yields (87% for 1i and 90% for 1j) (Scheme 3). Additionally, our optimization shows that the reaction system was significantly improved with CuCl as a catalyst with K_2_CO_3_ as base at 120 °C for styrene derivatives as a substrate with α-haloacetonitriles (α-bromo/α-iodo-acetonitrile) as cyanomethyl source (Scheme 4). In order to elaborate the scope of substrate, a variety of styrenes were examined to get the variety of β,γ-unsaturated nitriles products (Scheme 4). The electron donating substituted styrenes such as *p*-1-methyl-4-vinylbenzene (4a), 1-methoxy-4-vinylbenzene (4b), 1-(*tert*-butyl)-4-vinylbenzene (4c), 1-methoxy-2-vinylbenzene (4d) and 4-vinyl-1,1′-biphenyl (4e) were allowed 73% to 90% yields of β,γ-unsaturated cyanated products (5a–5e) (Scheme 4). Gratifyingly, prop-1-en-2-ylbenzene (4f) underwent into desired 4-phenylpent-3-enenitrile (5f) products with 69% yields and (1 : 1) *E*/*Z* (Scheme 4). Moreover, by using α-iodo-acetonitrile as cross coupling partner of the (1-bromovinyl)benzene (4g) to form 4-bromo-4-phenylbut-3-enenitrile (5g) in 70% yield (Scheme 4). To our surprise, 2-vinylbenzoic acid (4h) gave the corresponding product (*E*)-2-(3-cyanoprop-1-en-1-yl)benzoic acid (5h) product with high yield (71%) (Scheme 4). Remarkably, the reaction with 1,1-diphenylethylene (4i) worked well and afforded the target product (5i) with 94% yield (Scheme 4). Delightfully, when one non-fused ring (ethene-1,1-diyldibenzene) was installed with electron donating substituent methyl (4j), and electron withdrawing substituents (bromo and chloro) for β,γ-unsaturated products (5j, 5k, 5l) in good to excellent yields (81–88%) with (1 : 0.17 and 1 : 0.1) *E*/*Z* respectively. Additionally, both non-fused rings of ethene-1,1-diyldibenzene installed with electron donating substituent methyl (4m), methoxy (4n), and electron withdrawing substituents bromo (4o) and chloro (4p) have low impact on the reaction efficiency, resulting β,γ-unsaturated products (5m to 5p) with good to excellent yields (78–88%). Moreover, the product 3-(10,11-dihydro-5*H*-dibenzo[*a*,*d*][7]annulen-5-ylidene)propanenitrile (5q), mainly found in the biological active compounds, could synthesize in our reaction system by allowing cyanomethyl functionalization through a cross-coupling of 5-methylene-10,11-dihydro-5*H*-dibenzo[*a*,*d*][7]annulene (4q) and α-bromo-acetonitrile with 94% yield (Scheme 4).

**Scheme 5:**
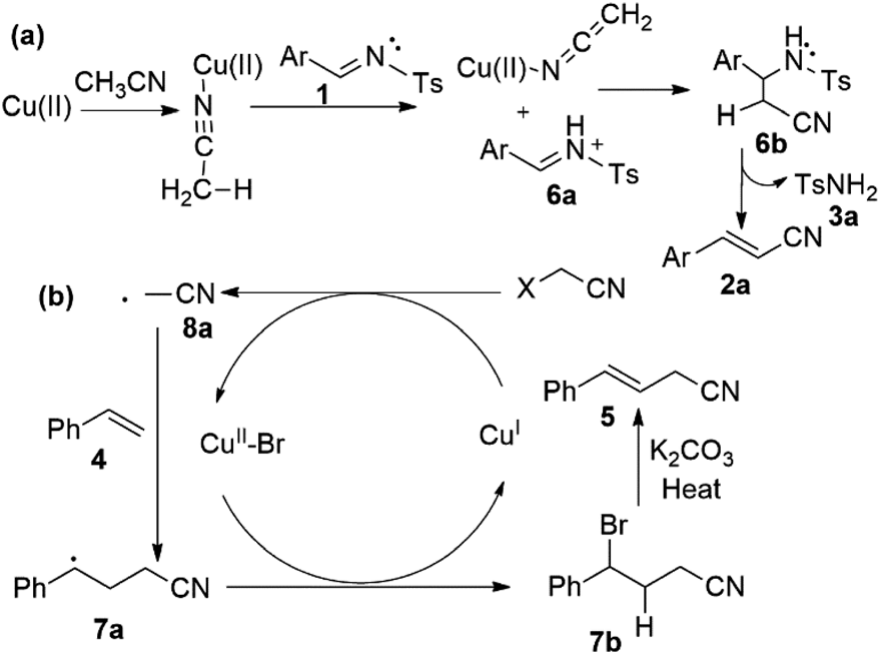
Proposed mechanism for Cu-catalyzed cyanomethylation of imines and styrenes.

On the basis of reported mechanistic studies^8,9^ we examined this reaction and propose a possible pathway of reaction as described in Scheme 5. Accordingly, the C(sp^3^)–H activation of acetonitrile was possibly promoted by copper species (Scheme 5a).^10^ Firstly, cyano of acetonitrile could coordinates with Cu species and speculate that the acetonitrile deprotonated *via* capture of proton by imine substrate (1) to generate nucleophile of acetonitrile (Scheme 5a). Further, it can coordinates with proposed 6a and produces 6b possible species (Scheme 5a). Moreover, 3a (methylbenzenesulfonamide) and 2a (phenylacrylonitrile) could be formed by dehydrogenation and recovered proton transferred to imine (Scheme 5a).

Similarly, bromo-acetonitrile activated by copper metal into radical species 8a (Scheme 5b). Consequently, substrate (4) was converted into 7a with Cu(i) species through single electron transfer (SET), was observed by adding 2 equivalent of TEMPO, which abstract a radical hydrogen to form TEMPOH. In addition, TEMPOCH_2_CN was isolated and confirmed by NMR and spectra was mentioned in ESI.†

Radical of acetonitrile (8a) coupled with substrate (4) and generated intermediate 7a (Scheme 5b). Additionally, this 7a could converted into 7b intermediate by bromide transfer from Cu(ii)–Br species (Scheme 5b). This kind of intermediate 7b–1 confirmed by NMR spectroscopy though performing the reaction using 1-chloro-3-vinylbenzene (4r) as substrate under our standard conditions and isolated 4-bromo-4-(3-chlorophenyl)butanenitrile (7b–1) (ESI).† The Cu(i) completed catalytic cycle and intermediate 7b or 7b–1 underwent elimination of proton and gave β,γ-unsaturated cyanomethylated product (5) in the presence of K_2_CO_3_ (Scheme 5b). Currently, further mechanstic study is ongoing in our laboratory.

## Conclusion

We report copper catalyzed cyanomethylation of imines and α,β-alkenes with acetonitrile (MeCN) and its derivatives for the synthesis of arylacrylonitriles and β,γ-unsaturated nitriles. Moreover, considering the importance of acrylonitrile and β,γ-unsaturated nitriles, this protocol has potential in the industrial production. This method could tolerates broad scope of substrate with substitution of variety of functional groups led good to excellent yields (69–98%). These aromatic acrylonitriles and β,γ-unsaturated nitriles has application in organic reactions and medicinal chemistry which founded in biologically active products.

## Conflicts of interest

There are no conflicts to declare.

## Supplementary Material

RA-011-D0RA10693C-s001

## References

[cit1] (a) Kleemann A. , Engel J., Kutscher B. and Reichert D., Pharmaceutical Substance: Synthesis, Patents, Applications, Georg Thieme, Stuttgart, Germany, 4th edn, 2001

[cit2] Anbarasan P., Schareina T., Beller M. (2011). Chem. Soc. Rev..

[cit3] Murakami M., Kato T., Mukaiyama T. (1987). Chem. Lett..

[cit4] Munemori D., Tsuji H., Uchida K., Suzuki T., Isa K., Minakawa M., Kawatsura M. (2014). Synthesis.

[cit5] Masui M., Yamagata K., Ueda C., Ohmori H. (1985). J. Chem. Soc., Chem. Commun..

[cit6] Abermil N., Masson G., Zhu J. (2008). J. Am. Chem. Soc..

[cit7] Xiao -F. W., Chloé V.-L., Bray L. B., Christophe D. (2009). Tetrahedron.

[cit8] Li Z., Bohle D. S., Li C.-J. (2006). Proc. Natl. Acad. Sci. U. S. A..

[cit9] Boess E., Sureshkumar D., Sud A., Wirtz C., Fars C., Klussmann M. (2011). J. Am. Chem. Soc..

[cit10] Lpez R., Palomo C. (2015). Angew. Chem..

