# Supplementary material for: Cu-catalyzed cyanomethylation of imines and α,β-alkenes with acetonitrile and its derivatives
Source: RSC Adv. 2021 Jan 28;11(10):5427–31. doi: 10.1039/d0ra10693c (PMC8694676; doi:10.1039/d0ra10693c)
Supplement: RA-011-D0RA10693C-s001 [file RA-011-D0RA10693C-s001.pdf]

Electronic Supplementary Material (ESI) for RSC Advances.
This journal is © The Royal Society of Chemistry 2021

Supporting Information for

Cu-catalyzed Cyanomethylation of Imines and α,β-alkenes with with Acetonitrile and its Derivatives

Muhammad Siddique Ahmad[a] and Atique Ahmad[b]*

[a]Institute of Chemical Sciences, Bahauddin Zakaryia University, Multan, Pakistan.
[b]Air University, Islamabad Campus, Pakistan
E-mail: doctormsahmad@gmail.com

Table of Contents

1. General procedure ……………………………………………………………………………………………..S1
2. Synthesis of (E)-N-benzylidene-4-methylbenzenesulfonamide substrate derivatives ……………………………………………………………………………………………………..S1
3. Reaction procedure for synthesis of aryl acrylonitriles from E)-N-benzylidene-4-methylbenzenesulfonamide derivatives..S3
4. Reaction procedure for synthesis of β,γ-unsaturated nitriles from α,β-alkenes derivatives..S3
5. Spectral information data…..S9
6. References S9
7. Copies of ^{1}H NMR spectra S10

1. General procedure

Experimental part: The reagents in the experiments were commercially available from the chemical company. All reagents were dried and distilled according to the standard reagent purification manual prior to use. The analyzed thin layer chromatography (TLC) was performed on a 60 F254 silica gel plate. TLC color development was developed by UV lamp (254 nm) and potassium permanganate. The ^{1}H NMR spectrum was obtained on a Mercury Plus-400(400 MHz). Chemical shifts are obtained as a residual solvent peak or trimethylsilane. Mercury Plus-400 (100 MHz) also obtained the ^{13}C NMR spectrum. The chemical shift was obtained by calibrating the intermediate peak of the deuterated chloroform triplet to 77.0 ppm or the deuterated dimethyl sulfoxide intermediate peak to 39.5 ppm. High-resolution mass spectrometry (HRMS) was measured at the ACQUITYTM UPLC & Q-TOF MS Premier instrument at the H.E.J., ICCBS, University of Karachi, Pakistan at Analytical Testing Center. Gas-mass chromatographic analysis was performed on a LECO Pegasus 4D GC x GC-TOFMS instrument. Gas chromatographic analysis was performed on a Shimadzu GC-2014 instrument.

2. Synthesis of (E)-N-benzylidene-4-methylbenzenesulfonamide substrate derivatives

All known substrates were prepared by followed the reported methodologies which mentioned in literature 1,2,3,4,5,6 and confirmed with NMR spectroscopy data reported.

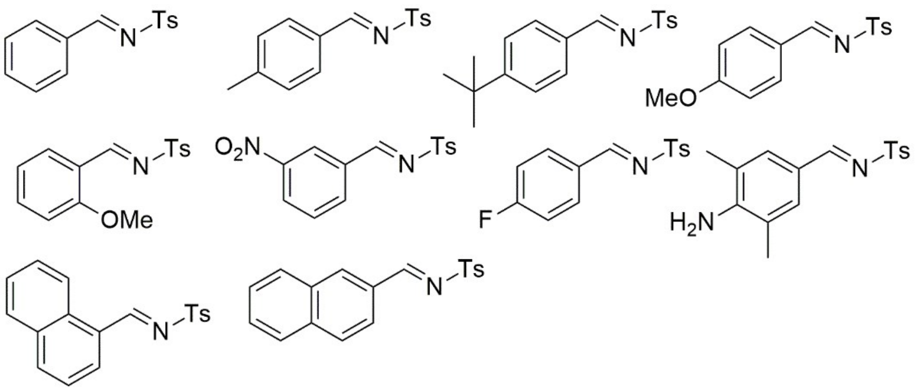

3. Reaction procedure for synthesis of aryl acrylonitriles from E)-N-benzylidene-4-methylbenzenesulfonamide derivatives

The $Cu(OAc)_2$ (0.04 mmol, 7.2 mg, 20 mol%) and (E)-N-benzylidene-4-methylbenzenesulfonamide (0.2 mmol) in acetonitrile (1.2 mL) were heated and stirred at 135 °C for 24 h into a Schlenk tube (25 mL) with a magnetic stir bar under N_2 atmosphere. The cooled solution was diluted and extracted with EA/H_2O and washed in sequence with brine solution. The organic layer was dried with Na_2SO_4 and concentrated in vacuo. The crude material was purified by flash silica chromatography to afford the corresponding aryl alkenyl cyanated product.

4. Reaction procedure for synthesis of β,γ-unsaturated nitriles from α,β-alkenes derivatives

The CuCl (0.04 mmol, 20 mol%) and α,β-alkenes (0.2 mmol) and K_2CO_3 (0.2 mmol, 1.0 eq.) with bromoacetonitrile (0.2 mmol, 1.0 eq.) in acetonitrile (1.2 mL) were heated and stirred at 120 °C for 24 h into a Schlenk tube (25 mL) with a magnetic stir bar under N_2 atmosphere. The cooled solution was diluted and extracted with EA/H_2O and washed in sequence with brine solution. The organic layer was dried with Na_2SO_4 and concentrated in vacuo. The crude material was purified by flash silica chromatography to afford the corresponding β,γ-unsaturated nitriles product.

5. Spectral information data

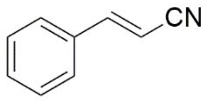

Cinnamonitrile (2a)

White solid (**2a**: 98% yields), ^{1}**H NMR** (400 MHz, Chloroform-*d*) δ (ppm): 7.46–7.38 (m, 6H), 5.88 (d, *J* ＝16.0 Hz, 1H). This compound was prepared using our procedures and spectra are consistent with literature.[7-11]

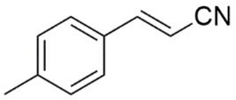

(*E*)-3-(*p*-tolyl)acrylonitrile (2b)

White liquid (**2b**: 80% yield); ^{1}**H NMR** (400 MHz, Chloroform-*d*) δ (ppm): 7.38-7.34 (m, 3H), 7.21 (d, *J*= 8.0 Hz, 2H), 5.82 (d, *J* = 16.0 Hz, 2H), 2.38 (s, 1H). This compound was prepared using our procedure and spectra are consistent with literature. [7-11]

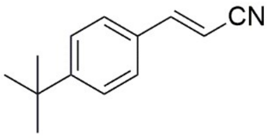

(*E*)-3-(4-(*tert*-butyl)phenyl)acrylonitrile (2c)

White liquid (**2c**: 75% yield); ^{1}**H NMR** (400 MHz, Chloroform-*d*) δ (ppm): 7.42–7.40 (m, 5H), 5.84 (d, *J* = 16.0 Hz, 1H), 1.33 (s, 9H). This compound was prepared using our general procedure and spectra are consistent with literature. [7-11]

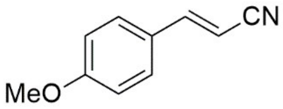

(*E*)-3-(4-methoxyphenyl)acrylonitrile (2d)

White solid (**2d**: 84% yields), ^{1}**H NMR** (400 MHz, Chloroform-*d*) δ (ppm): 7.40 (d, *J* = 8.0 Hz, 2H), 7.34 (d, *J*= 16.0 Hz, 1H), 6.92 (d, *J* = 8.0 Hz, 1H), 5.72 (d, *J* =16.0 Hz, 2H), 3.85 (s, 1H). This compound was prepared using our procedure and spectra are consistent with literature.[7-11]

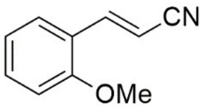

(*E*)-3-(2-methoxyphenyl)acrylonitrile (2e)

White liquid (**2e**: 88% yield); ^{1}**H NMR** (400 MHz, Chloroform-*d*) δ (ppm): 7.63 (d, *J* = 16.0 Hz, 1H), 7.38 (d, *J* = 8.0 Hz, 2H), 6.94 (t, J_1 = 8.0 Hz, J_2 = 12.0 Hz, 2H), 6.06 (d, *J*= 16.0 Hz, 1H), 3.90 (s, 3H). This compound was prepared using our general procedure and spectra are consistent with literature. [7-11]

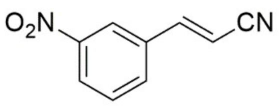

(*E*)-3-(3-nitrophenyl)acrylonitrile (2f)

Colorless liquid (**2f**: 69% yield); **^{1}H NMR** (400 MHz, Chloroform-*d*) δ 7.48 (dd, *J* = 8.7, 5.4 Hz, 2H), 7.39 (d, *J* = 16.6 Hz, 1H), 7.13 (t, *J* = 8.6 Hz, 2H), 5.83 (d, *J* = 16.7 Hz, 1H). This compound was prepared using our general procedure and spectra are consistent with literature. [7-11]

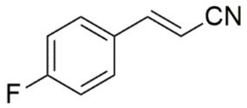

(*E*)-3-(4-fluorophenyl)acrylonitrile (2g)

Colorless liquid (**2g**: 71% yield); **^{1}H NMR** (400 MHz, Chloroform-*d*) δ 7.48 (dd, *J* = 8.7, 5.4 Hz, 2H), 7.39 (d, *J* = 16.6 Hz, 1H), 7.13 (t, *J* = 8.6 Hz, 2H), 5.83 (d, *J* = 16.7 Hz, 1H). This compound was prepared using our general procedure and spectra are consistent with literature. [7-11]

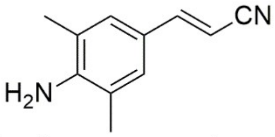

(*E*)-3-(4-amino-3,5-dimethylphenyl)acrylonitrile (2h)

White solid (**2h**: 87% yield); **^{1}H NMR** (400 MHz, Chloroform-*d*) δ 7.28 (d, *J* = 1.0 Hz, 1H), 7.08 (s, 2H), 5.62 (d, *J* = 16.5 Hz, 1H), 4.06 – 3.88 (m, 2H), 2.20 (s, 6H). This compound was prepared using our general procedure and spectra are consistent with literature. [7-11]

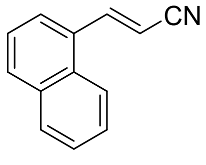

(*E*)-3-(naphthalen-1-yl)acrylonitrile (2i)

White solid (**2i**: 87% yield); **^{1}H NMR** (400 MHz, Chloroform-*d*) δ 8.28 (d, *J* = 16.4 Hz, 1H), 8.09 (d, *J* = 8.4 Hz, 1H), 7.95 (dd, *J* = 20.4, 8.2 Hz, 2H), 7.71 (d, *J* = 7.2 Hz, 1H), 7.62 (ddd, *J* = 13.6, 8.1, 1.5 Hz, 2H), 7.55 – 7.48 (m, 1H), 6.02 (d, *J* = 16.4 Hz, 1H). This compound was prepared using our general procedure and spectra are consistent with literature. [7-11]

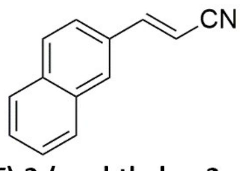

(*E*)-3-(naphthalen-2-yl)acrylonitrile (2j)

White solid (**2j**: 87% yield), **^{1}H NMR** (400 MHz, Chloroform-*d*) δ (ppm): 7.86–7.84 (m, 4H), 7.57–7.55 (m, 4H), 5.98 (d, *J*=16.0 Hz, 1H). This compound was prepared using our procedure and spectra are consistent with literature. [7-11]

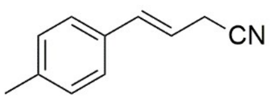

(*E*)-4-(*p*-tolyl)but-3-enenitrile (5a)

White solid (**5a**: 79% yield), **^{1}H NMR** (500 MHz, Chloroform-*d*) δ 7.21 (d, *J* = 6.4 Hz, 2H), 7.08 (d, *J* = 8.0 Hz, 2H), 6.64 (d, *J* = 15.8 Hz, 1H), 5.93 (dd, *J* = 15.8, 5.7 Hz, 1H), 3.22 (dd, *J* = 5.7, 1.7 Hz, 2H), 2.29 (s, 3H). This compound was prepared using our procedure and spectra are consistent with literature.[12]

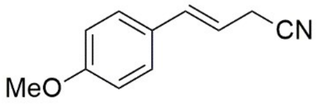

(*E*)-4-(4-methoxyphenyl)but-3-enenitrile (5b)

White solid (**5b**: 89% yield), **^{1}H NMR** (500 MHz, Chloroform-*d*) δ 7.32 (d, *J* = 8.7 Hz, 2H), 6.88 (d, *J* = 8.8 Hz, 2H), 6.68 (d, *J* = 15.8 Hz, 1H), 5.92 (dt, *J* = 15.8, 5.7 Hz, 1H), 3.83 (s, 3H), 3.28 (s, 2H). This compound was prepared using our procedure and spectra are consistent with literature.[12]

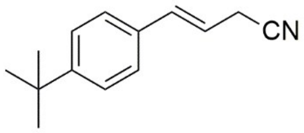

(*E*)-4-(4-(*tert*-butyl)phenyl)but-3-enenitrile (5c)

White liquid (**5c**: 78% yield), **^{1}H NMR** (400 MHz, Chloroform-*d*) δ 7.39 – 7.29 (m, 4H), 6.72 (d, *J* = 15.7 Hz, 1H), 6.02 (dt, *J* = 15.8, 5.7 Hz, 1H), 3.29 (dd, *J* = 5.7, 1.7 Hz, 2H), 1.33 (s, 9H). This compound was prepared using our procedure and spectra are consistent with literature.[12]

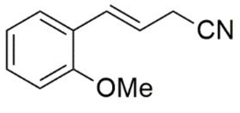

(*E*)-4-(2-methoxyphenyl)but-3-enenitrile (5d)

White liquid (**5d**: 73% yield), **^{1}H NMR** (500 MHz, Chloroform-*d*) δ 7.30 (dd, *J* = 7.6, 1.5 Hz, 1H), 6.93 (d, *J* = 15.9 Hz, 1H), 6.86 (t, *J* = 7.5 Hz, 1H), 6.82 (d, *J* = 8.2 Hz, 1H), 6.04 (dt, *J* = 15.9, 5.9 Hz, 1H), 3.79 (s, 3H), 3.23 (dd, *J* = 5.9, 1.7 Hz, 2H). This compound was prepared using our procedure and spectra are consistent with literature.[12]

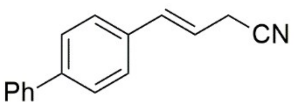

(*E*)-4-([1,1'-biphenyl]-4-yl)but-3-enenitrile (5e)

White liquid (**5e**: 90% yield), **^{1}H NMR** (400 MHz, Chloroform-*d*) δ 7.63 – 7.57 (m, 4H), 7.46 (dt, *J* = 7.8, 3.3 Hz, 4H), 7.37 (d, *J* = 7.4 Hz, 1H), 6.79 (d, *J* = 15.8 Hz, 1H), 6.11 (dt, *J* = 15.8, 5.7 Hz,

1H), 3.33 (dd, *J* = 5.7, 1.7 Hz, 2H). This compound was prepared using our procedure and spectra are consistent with literature.[12]

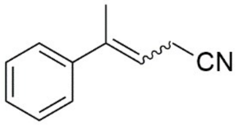

4-phenylpent-3-enenitrile (5f)

Colorless liquid (**5f**: 69% yield), **^{1}H NMR** (500 MHz, Chloroform-*d*) δ 7.36 (dd, *J* = 13.9, 6.3 Hz, 13H), 5.72 (t, *J* = 7.0 Hz, 1H), 5.42 (s, 1H), 5.22 (s, 1H), 3.26 (d, *J* = 7.8 Hz, 2H), 2.87 (t, *J* = 7.9 Hz, 2H), 2.48 (t, *J* = 7.4 Hz, 2H), 2.11 (s, 3H). This compound was prepared using our procedure and spectra are consistent with literature.[12]

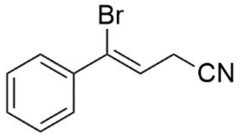

(*Z*)-4-bromo-4-phenylbut-3-enenitrile (5g)

Colorless liquid (**5g**: 70% yield), **^{1}H NMR** (400 MHz, Chloroform-*d*) δ 7.54 (d, *J* = 9.6 Hz, 2H), 7.38 (d, *J* = 4.1 Hz, 3H), 6.24 (t, *J* = 6.6 Hz, 1H), 3.46 (d, *J* = 6.6 Hz, 2H). This compound was prepared using our procedure and spectra are consistent with literature.[12]

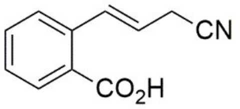

(*E*)-2-(3-cyanoprop-1-en-1-yl)benzoic acid (5h)

Brown solid (**5h**: 71% yield), **^{1}H NMR** (400 MHz, Chloroform-*d*) δ 8.02 (dd, *J* = 7.7, 1.1 Hz, 1H), 7.71 – 7.61 (m, 2H), 7.53 (dd, *J* = 17.4, 11.0 Hz, 1H), 7.46 – 7.41 (m, 1H), 5.75 (dd, *J* = 17.4, 1.2 Hz, 1H), 5.49 (dd, *J* = 11.0, 1.2 Hz, 1H), 5.01 (s, 2H). This compound was prepared using our procedure and spectra are consistent with literature.[12]

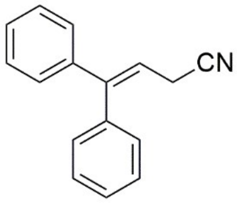

4,4-diphenylbut-3-enenitrile (5i)

White solid (**5i**: 94% yield), **^{1}H NMR** (400 MHz, Chloroform-*d*) δ 7.39 – 7.32 (m, 3H), 7.25 – 7.23 (m, 3H), 7.16 (dd, *J* = 6.8, 3.0 Hz, 2H), 7.14 – 7.10 (m, 2H), 5.98 (t, *J* = 7.4 Hz, 1H), 3.09 (d, *J* = 7.4 Hz, 2H). This compound was prepared using our procedure and spectra are consistent with literature.[13]

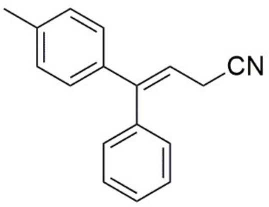

(*E*)-4-phenyl-4-(*p*-tolyl)but-3-enenitrile (5j)

Colorless liquid (**5j**: 90% yield), **^{1}H NMR** (400 MHz, Chloroform-*d*) δ 7.45 – 7.37 (m, 2H), 7.30 (dd, *J* = 4.5, 2.3 Hz, 1H), 7.23 (d, *J* = 7.3 Hz, 2H), 7.18 (dd, *J* = 8.0, 1.4 Hz, 1H), 7.12 (s, 3H), 7.07 (d, *J* = 8.0 Hz, 1H), 6.01 (t, *J* = 7.4 Hz, 1H), 3.15 (dd, *J* = 12.1, 7.4 Hz, 2H), 2.38 (d, *J* = 24.0 Hz, 3H). This compound was prepared using our procedure and spectra are consistent with literature.[13]

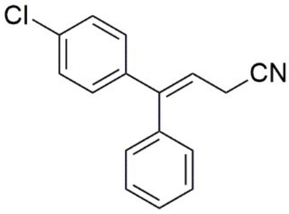

(*E*)-4-(4-chlorophenyl)-4-phenylbut-3-enenitrile (5k)

Colorless liquid (**5k**: 84% yield), **^{1}H NMR** (400 MHz, Chloroform-*d*) δ 7.50 (dd, *J* = 6.0, 3.3 Hz, 1H), 7.36 (dd, *J* = 5.9, 3.4 Hz, 2H), 7.31 (dd, *J* = 5.3, 1.9 Hz, 3H), 7.22 (dt, *J* = 6.5, 3.2 Hz, 3H), 6.20 (t, *J* = 7.2 Hz, 1H), 5.80 (t, *J* = 7.4 Hz, 0H), 3.32 (d, *J* = 7.4 Hz, 0H), 3.12 – 2.93 (m, 2H). This compound was prepared using our procedure and spectra are consistent with literature.[13]

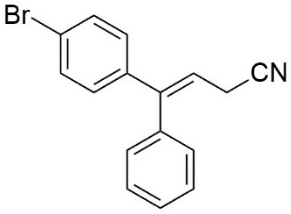

(*E*)-4-(4-bromophenyl)-4-phenylbut-3-enenitrile (5l)

Colorless liquid (**5l**: 88% yield), **^{1}H NMR** (400 MHz, Chloroform-*d*) δ 7.69 (dd, *J* = 8.0, 1.0 Hz, 1H), 7.41 (td, *J* = 7.5, 1.1 Hz, 2H), 7.31 (dt, *J* = 3.9, 2.0 Hz, 4H), 7.25 – 7.21 (m, 3H), 6.22 – 6.17 (m, 1H), 5.78 (d, *J* = 7.4 Hz, 0H), 3.34 (d, *J* = 7.4 Hz, 0H), 3.12 – 2.92 (m, 2H). This compound was prepared using our procedure and spectra are consistent with literature.[13]

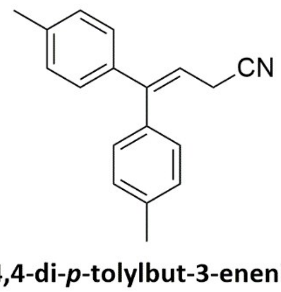

4,4-di-*p*-tolylbut-3-enenitrile (5m)

Colorless liquid (**5m**: 88% yield), **^{1}H NMR** (400 MHz, Chloroform-*d*) δ 7.25 (d, *J* = 7.9 Hz, 2H), 7.16 – 7.05 (m, 6H), 5.99 (t, *J* = 7.4 Hz, 1H), 3.16 (d, *J* = 7.4 Hz, 2H), 2.39 (d, *J* = 23.7 Hz, 6H). This compound was prepared using our procedure and spectra are consistent with literature.[13]

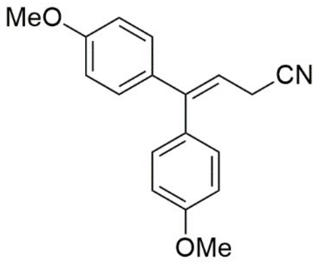

4,4-bis(4-methoxyphenyl)but-3-enenitrile (5n)

White solid (**5n**: 84% yield), **^{1}H NMR** (400 MHz, Chloroform-*d*) δ 7.16 (d, *J* = 8.8 Hz, 2H), 7.09 (d, *J* = 8.7 Hz, 2H), 6.94 (d, *J* = 6.6 Hz, 2H), 6.83 (d, *J* = 8.8 Hz, 2H), 5.89 (t, *J* = 7.4 Hz, 1H), 3.85 (s, 3H), 3.81 (s, 3H), 3.15 (d, *J* = 7.4 Hz, 2H). This compound was prepared using our procedure and spectra are consistent with literature.[13]

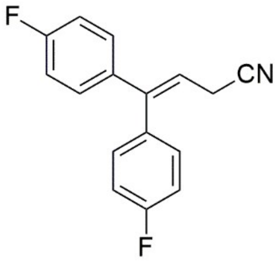

4,4-bis(4-fluorophenyl)but-3-enenitrile (5o)

Colorless liquid (**5o**: 79% yield), **^{1}H NMR** (400 MHz, Chloroform-*d*) δ 7.16 (td, *J* = 8.3, 7.8, 2.5 Hz, 6H), 7.01 (t, *J* = 8.6 Hz, 2H), 5.99 (t, *J* = 7.4 Hz, 1H), 3.14 (d, *J* = 7.4 Hz, 2H). This compound was prepared using our procedure and spectra are consistent with literature. [13]

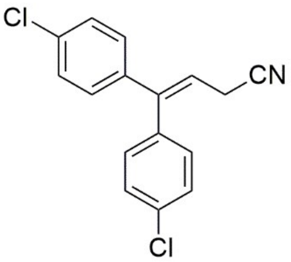

4,4-bis(4-chlorophenyl)but-3-enenitrile (5p)

Colorless liquid (**5p**: 78% yield), **^{1}H NMR** (400 MHz, Chloroform-*d*) δ 7.43 (d, *J* = 8.5 Hz, 2H), 7.33 – 7.29 (m, 2H), 7.13 (t, *J* = 8.8 Hz, 4H), 6.04 (t, *J* = 7.4 Hz, 1H), 3.14 (d, *J* = 7.4 Hz, 2H). This compound was prepared using our procedure and spectra are consistent with literature.[13]

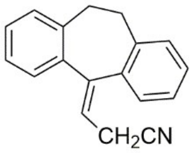

3-(10,11-dihydro-5*H*-dibenzo[*a*,*d*][7]annulen-5-ylidene)propanenitrile (5q)

Colorless solid (**5q**: 94% yield), **^{1}H NMR** (400 MHz, Chloroform-d) δ 7.29 (d, *J* = 7.2, Hz, 1H), 7.25 – 7.15(m, 5H), 7.12 (s, 1H), 7.08–7.06 (m, 1H), 5.86 (t, *J* = 7.4 Hz, 1H), 3.34 (t, *J* = 17.5 Hz, 2H), 3.16 (dd, *J* = 18.6, 7.3 Hz, 2H), 2.96 (t, *J* = 13.9 Hz, 1H), 2.83–2.81 (m, 1H). This compound was prepared using our procedure and spectra are consistent with literature. [13]

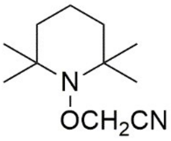

2-((2,2,6,6-tetramethylpiperidin-1-yl)oxy)acetonitrile

Colorless oil, 45% yield 10h. **^{1}H NMR** (400 MHz, Chloroform-*d*) δ 4.52 (s, 2H), 1.61 – 1.52 (m, 1H), 1.43 (d, *J* = 3.9 Hz, 1H), 1.38 – 1.24 (m, 2H), 1.20 (d, *J* = 3.9 Hz, 6H), 1.11 (d, *J* = 3.8 Hz, 6H). ^{1}H NMR of this known compound was matched with literature.[13]

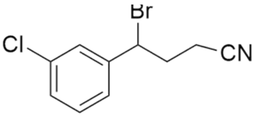

4-bromo-4-(3-chlorophenyl)butanenitrile (7b-1)

Colorless oil, 15% yield 13h. **^{1}H NMR** (500 MHz, Chloroform-d) δ 7.40 (s, 1H), 7.32 (dd, *J* = 4.0, 1.3 Hz, 2H), 7.30–7.28 (m, 1H), 4.98 (dd, *J* = 8.3, 5.8 Hz, 1H), 2.62–2.50 (m, 3H), 2.43–2.36 (m, 1H). ^{1}H NMR of this known compound was matched with literature.[12]

6. References

1. Xiao-F. W, Chloe´ V-L, Bray, L. B, Christophe D, *Tetrahedron* 2009, ***65***, 7380.
2. María D-G and Brian T. C. *Tetrahedron* 2011, ***67***, 7901.
3. Jessica Z. C. Wenzhi Y. Brian T. H. Charles K. L. and Masayuki W. *Angew. Chem. Int. Ed.* 2016, ***55***, 13877.
4. Hao C. Yu Z. Dong Z. Jinyi X. and Hong L. *Chem. Commun.,* 2014, ***50***, 14771.
5. Manas K. G. Subhomoy D. Kalpataru D. and Amit K. *Org. Biomol. Chem*., 2015, ***13,*** 9042; Ana C. C. Alberto F. and Jos A. *Chem. Eur. J.* 2018, ***24***, 3117.
6. Mar Fr. Abdolkarim Z. Ahmad R. M-Z. Alireza H. Abolfath P. Ali Khalafi-N. and Mohammad H. B. *Synth. Commun*. 2009, ***39***, 3156.
7. Zhang, G.; Zhang, L.; Hu, M.; Cheng, J. *Adv. Synth. Catal.* 2011, ***353***, 29.
8. Anbarasan, P.; Neumann, H.; Beller, M. *Angew.Chem. Int. Ed.* 2011, ***50****, 519*.
9. Zhang, Song-L.; Deng, Zhu-Q. *Org.Biomol. Chem.* 2016, ***14,*** 7282.

10. Pradal, A.; Evano, G. *Chem. Comm*. 2014, ***50***, 11907.

11. Ye, F.; Chen, J.; Ritter, T. *J. Am. Chem. Soc*. 2017, ***139***, 7184.

12. Pu, W. Y.; Sun, D.; Fan, W. Y.; Pan, W. W.; Chai, Q. H.; Wang, X. X.; Lv, Y. H.. *Chem. Commun.* 2019, ***55***, 4821.

13. Saisai, Z. Zengming, S. Hong, J. *J. Org. Chem.* 2020, ***9***, 6143.

7. Copies of ^{1}H NMR spectra

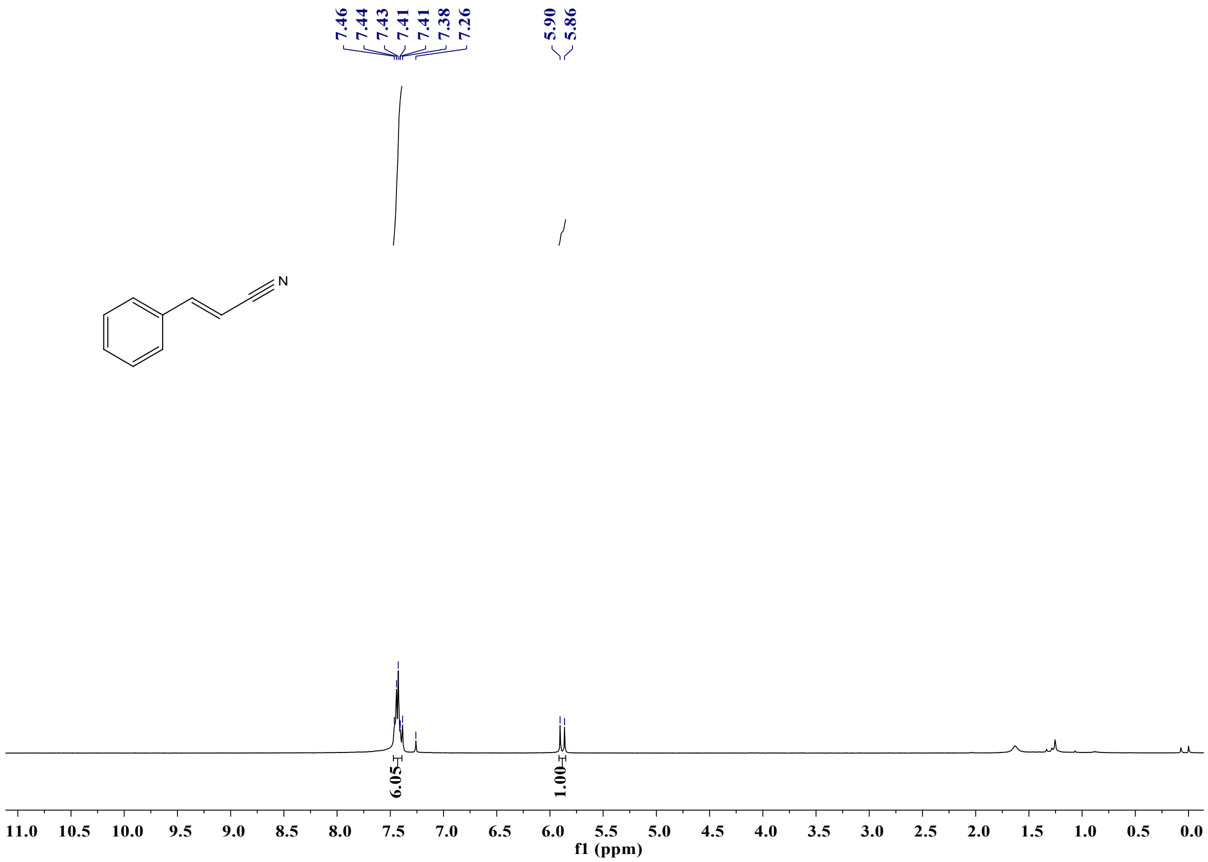

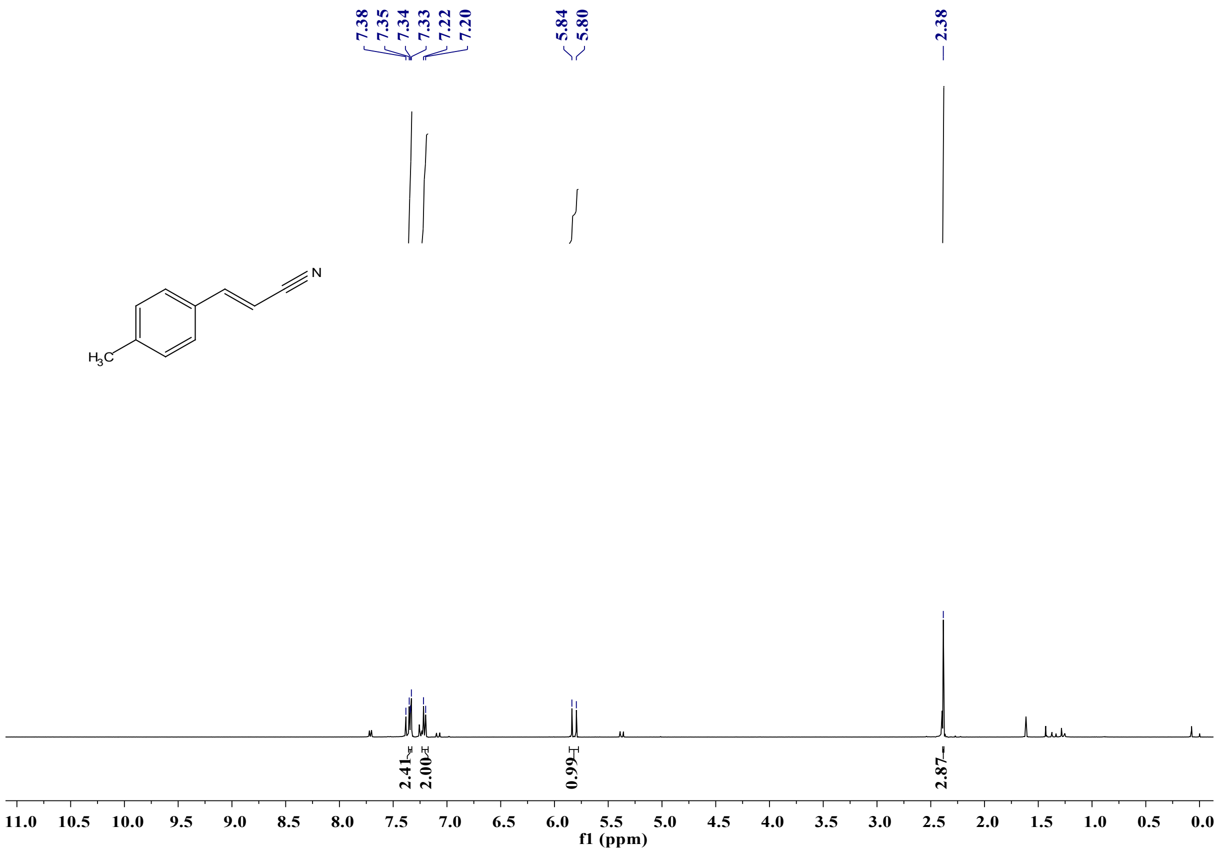

7.38
7.35
7.34
7.33
7.22
7.20
5.84
5.80
2.38
N
H3C
2.41
2.00
0.99
2.87
11.0 10.5 10.0 9.5 9.0 8.5 8.0 7.5 7.0 6.5 6.0 5.5 5.0 4.5 4.0 3.5 3.0 2.5 2.0 1.5 1.0 0.5 0.0
f1 (ppm)

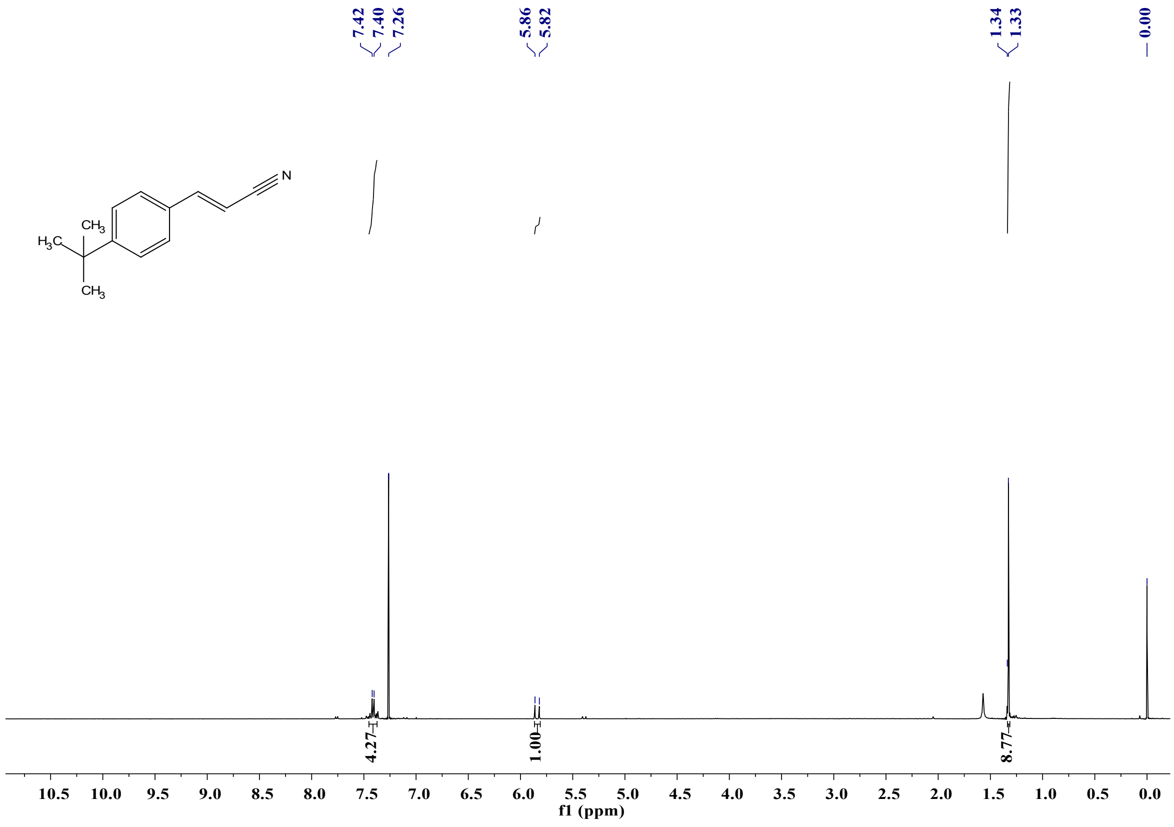

7.42
7.40
7.26
5.86
5.82
1.34
1.33
0.00
N
CH3
H3C
CH3
4.27
1.00
8.77
10.5 10.0 9.5 9.0 8.5 8.0 7.5 7.0 6.5 6.0 5.5 5.0 4.5 4.0 3.5 3.0 2.5 2.0 1.5 1.0 0.5 0.0
f1 (ppm)

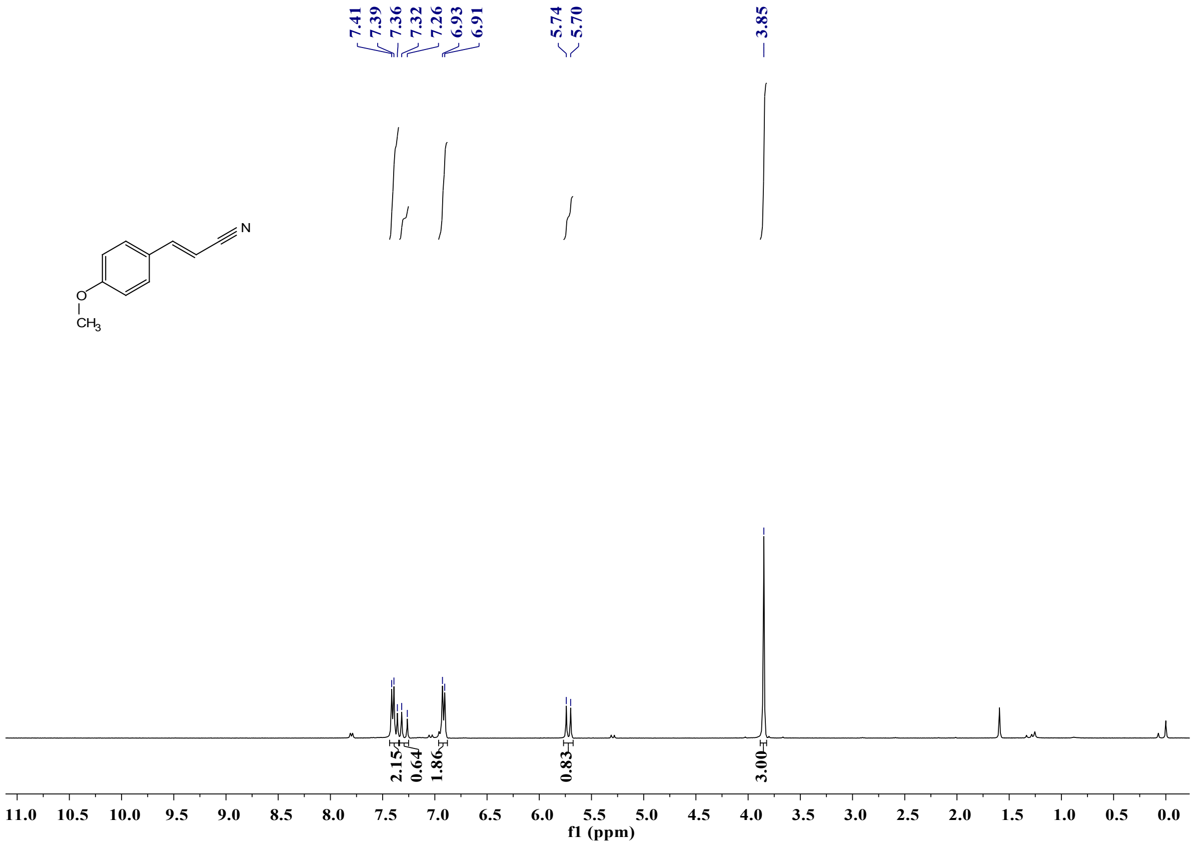

7.41
7.39
7.36
7.32
7.26
6.93
6.91
5.74
5.70
3.85
N
O
CH3
2.15
0.64
1.86
0.83
3.00
11.0 10.5 10.0 9.5 9.0 8.5 8.0 7.5 7.0 6.5 6.0 5.5 5.0 4.5 4.0 3.5 3.0 2.5 2.0 1.5 1.0 0.5 0.0
f1 (ppm)

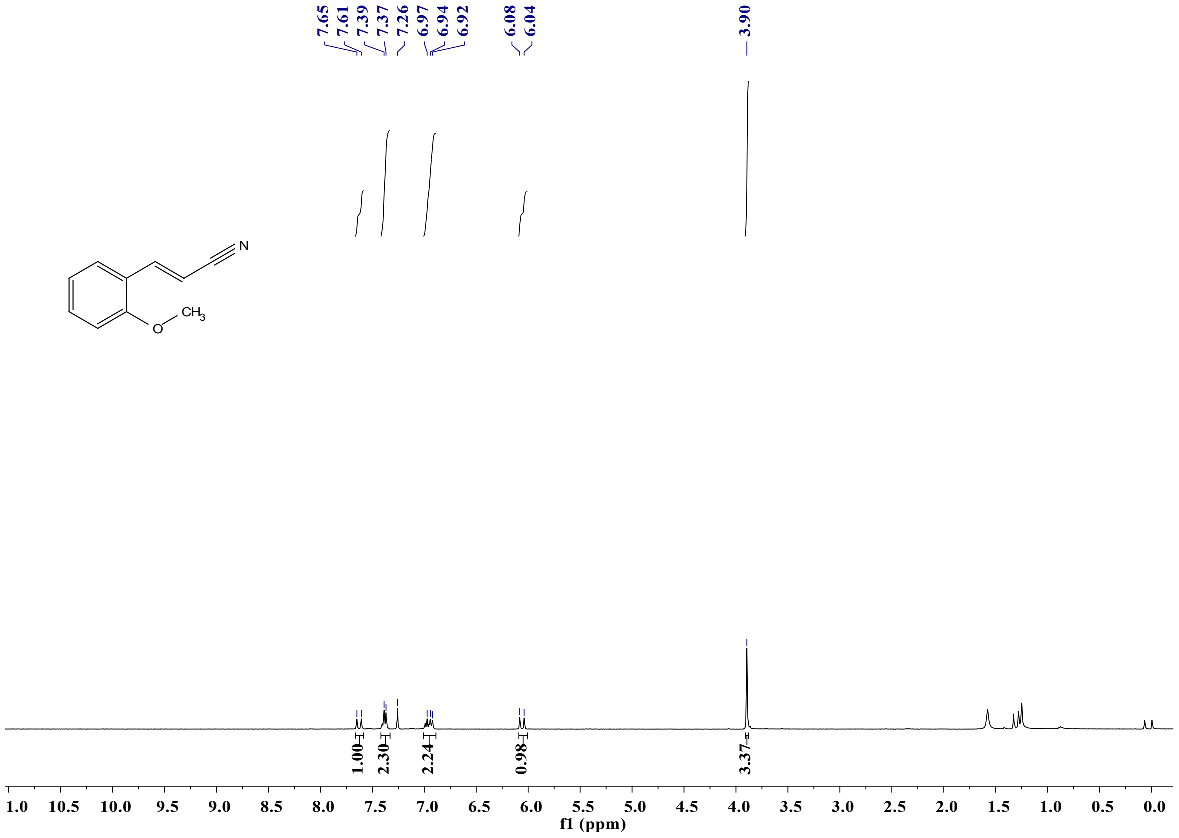

7.65
7.61
7.39
7.37
7.26
6.97
6.94
6.92
6.08
6.04
3.90
N
O
CH3
1.00
2.30
2.24
0.98
3.37
1.0 10.5 10.0 9.5 9.0 8.5 8.0 7.5 7.0 6.5 6.0 5.5 5.0 4.5 4.0 3.5 3.0 2.5 2.0 1.5 1.0 0.5 0.0
f1 (ppm)

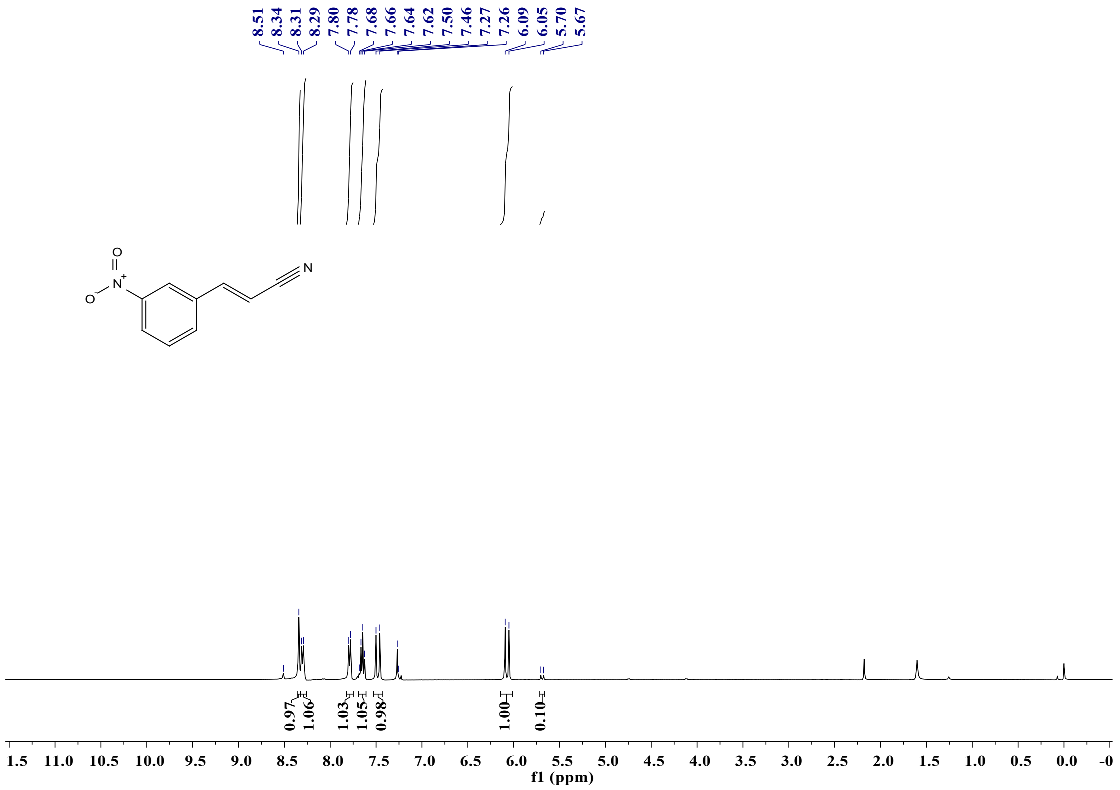
8.51
8.34
8.31
8.29
7.80
7.78
7.68
7.66
7.64
7.62
7.50
7.46
7.27
7.26
6.09
6.05
5.70
5.67
0.97
1.06
1.03
1.05
0.98
1.00
0.10
f1 (ppm)

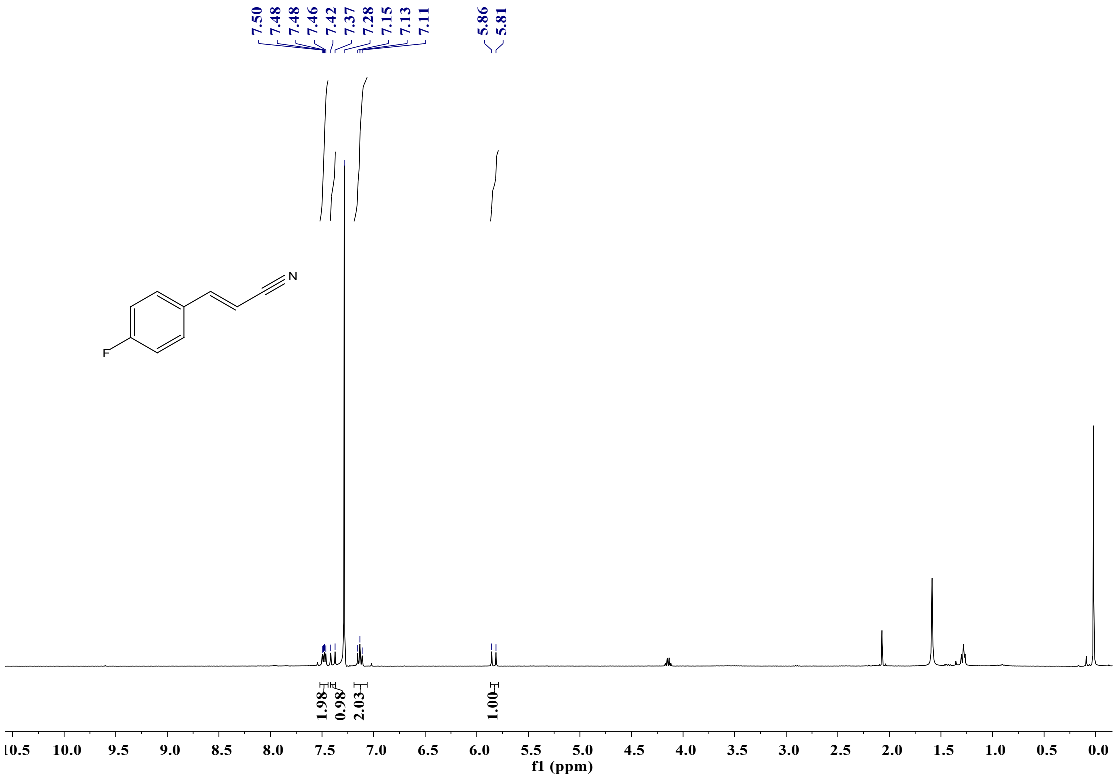
7.50
7.48
7.48
7.46
7.42
7.37
7.28
7.15
7.13
7.11
5.86
5.81
F
N
1.98
0.98
2.03
1.00
f1 (ppm)

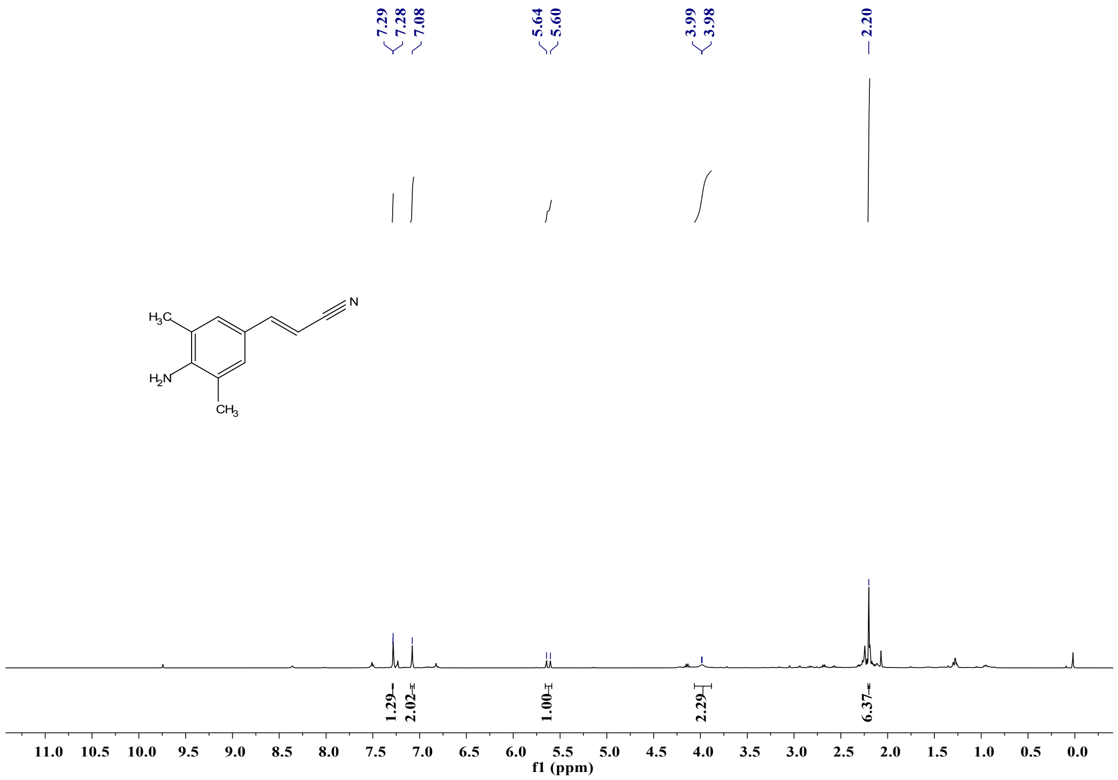
7.29
7.28
7.08
5.64
5.60
3.99
3.98
2.20
H3C
H2N
CH3
N
1.29
2.02
1.00
2.29
6.37
11.0 10.5 10.0 9.5 9.0 8.5 8.0 7.5 7.0 6.5 6.0 5.5 5.0 4.5 4.0 3.5 3.0 2.5 2.0 1.5 1.0 0.5 0.0
f1 (ppm)

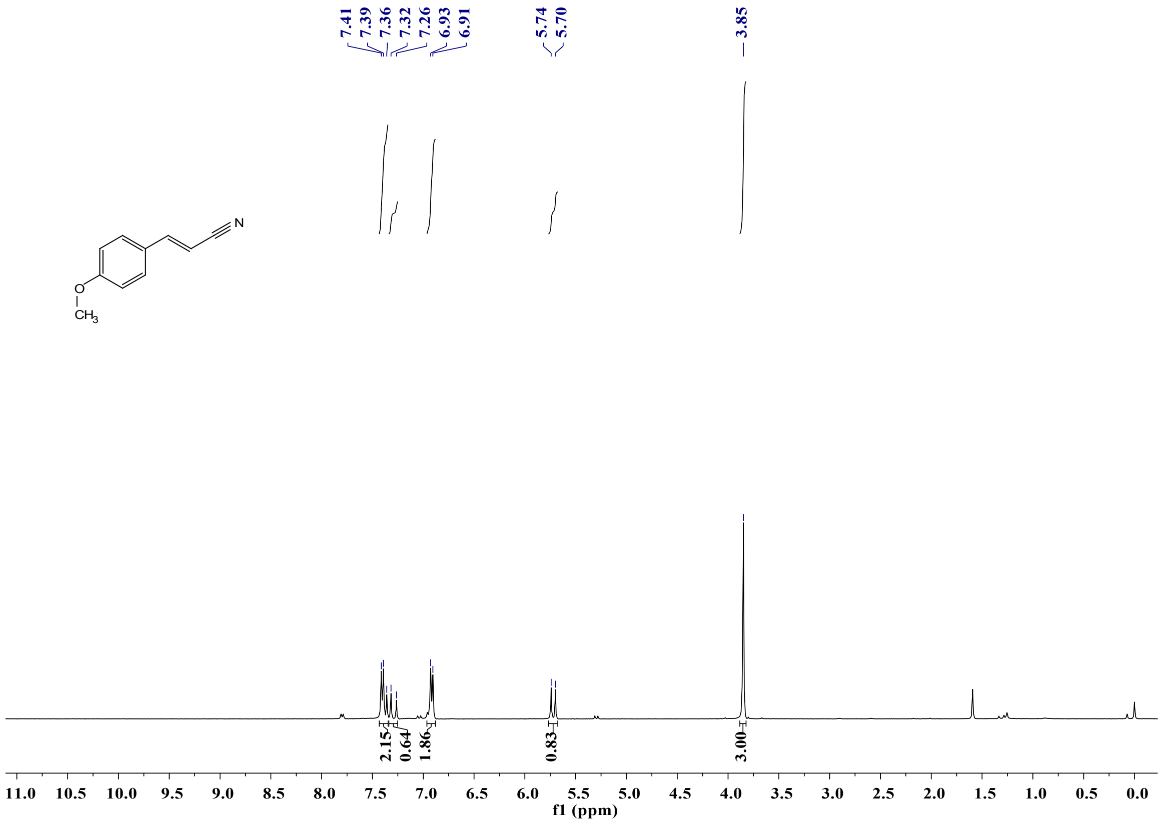
7.41
7.39
7.36
7.32
7.26
6.93
6.91
5.74
5.70
3.85
N
O
CH3
2.15
0.64
1.86
0.83
3.00
11.0 10.5 10.0 9.5 9.0 8.5 8.0 7.5 7.0 6.5 6.0 5.5 5.0 4.5 4.0 3.5 3.0 2.5 2.0 1.5 1.0 0.5 0.0
f1 (ppm)

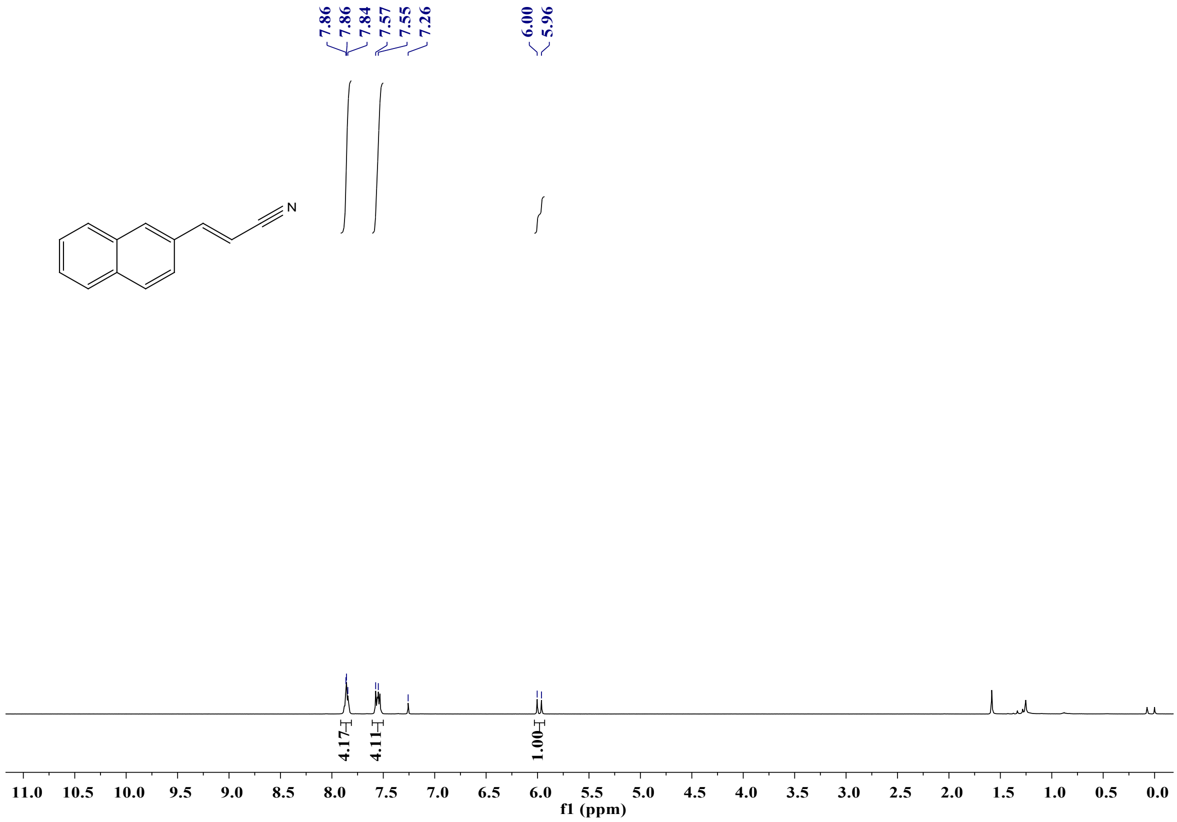

7.86
7.86
7.84
7.57
7.55
7.26
6.00
5.96
N
4.17
4.11
1.00
11.0 10.5 10.0 9.5 9.0 8.5 8.0 7.5 7.0 6.5 6.0 5.5 5.0 4.5 4.0 3.5 3.0 2.5 2.0 1.5 1.0 0.5 0.0
f1 (ppm)

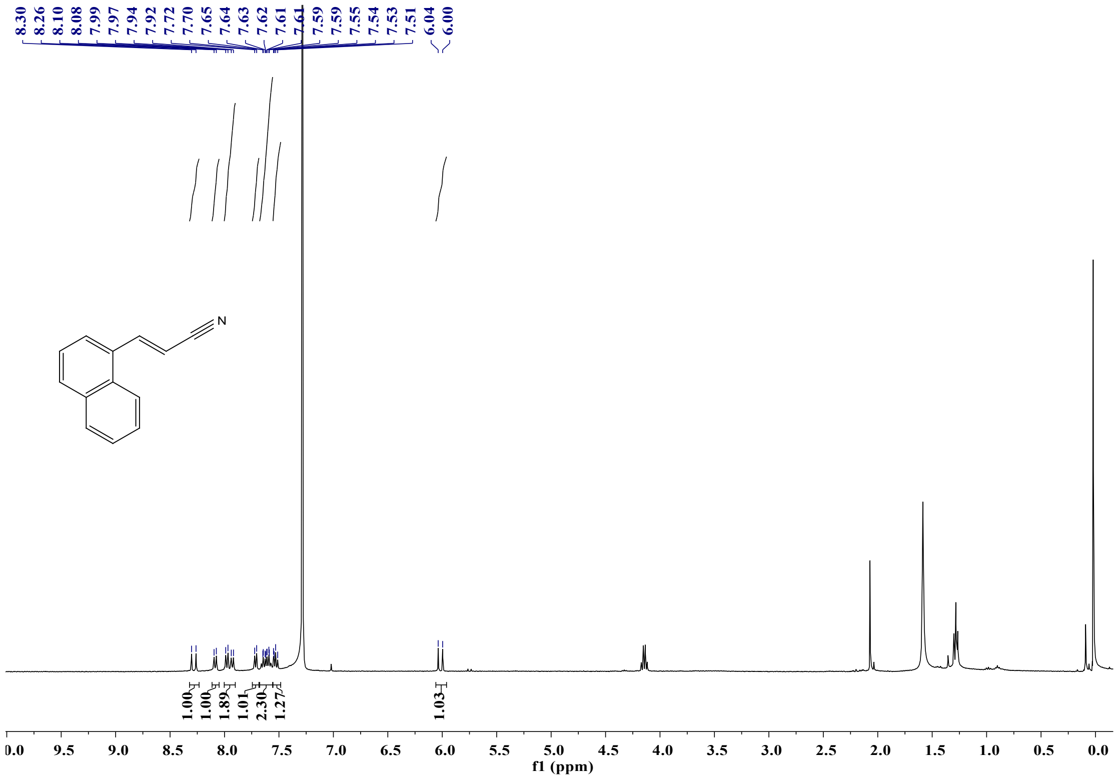

8.30
8.26
8.10
8.08
7.99
7.97
7.94
7.92
7.72
7.70
7.65
7.64
7.63
7.62
7.61
7.61
7.59
7.59
7.55
7.54
7.53
7.51
6.04
6.00
N
1.00
1.00
1.89
1.01
2.30
1.27
1.03
0.0 9.5 9.0 8.5 8.0 7.5 7.0 6.5 6.0 5.5 5.0 4.5 4.0 3.5 3.0 2.5 2.0 1.5 1.0 0.5 0.0
f1 (ppm)

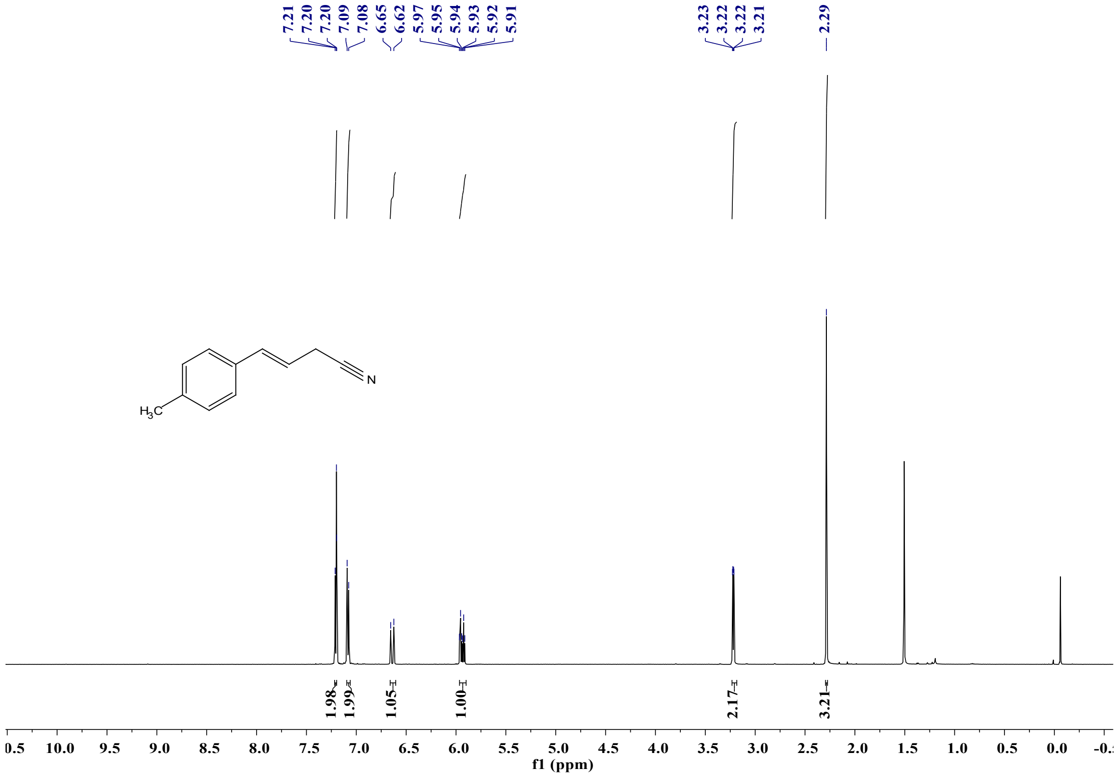
7.21 7.20 7.20 7.09 7.08 6.65 6.62 5.97 5.95 5.94 5.93 5.92 5.91
3.23 3.22 3.22 3.21
2.29
H3C
N
1.98 1.99 1.05 1.00 2.17 3.21
f1 (ppm)

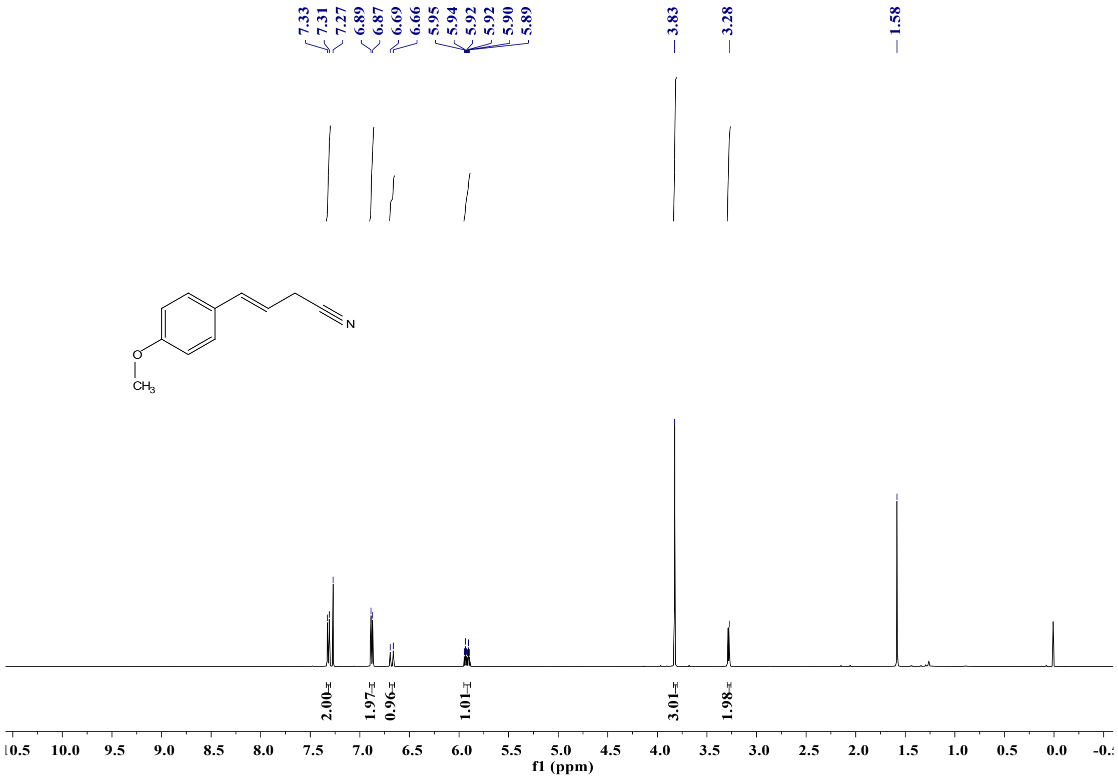
7.33 7.31 7.27 6.89 6.87 6.69 6.66 5.95 5.94 5.92 5.92 5.90 5.89
3.83
3.28
1.58
O
CH3
N
2.00 1.97 0.96 1.01 3.01 1.98
f1 (ppm)

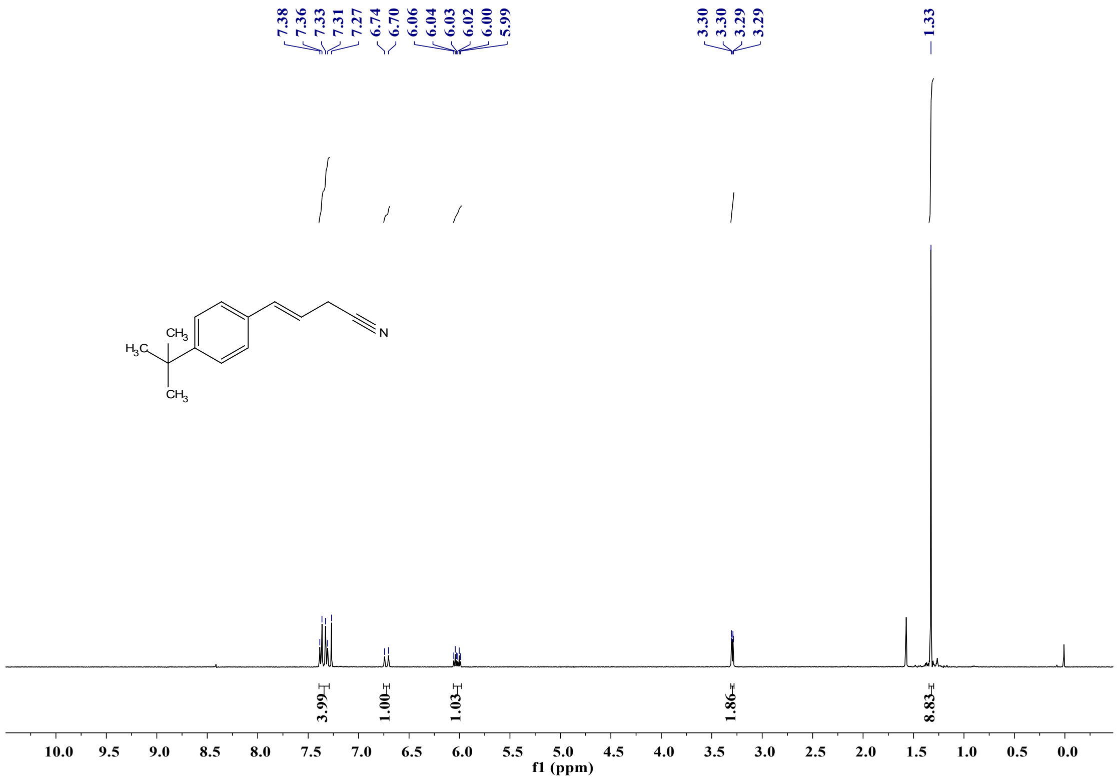
7.38
7.36
7.33
7.31
7.27
6.74
6.70
6.06
6.04
6.03
6.02
6.00
5.99
3.30
3.30
3.29
3.29
1.33
CH3
H3C
CH3
N
3.99
1.00
1.03
1.86
8.83
10.0 9.5 9.0 8.5 8.0 7.5 7.0 6.5 6.0 5.5 5.0 4.5 4.0 3.5 3.0 2.5 2.0 1.5 1.0 0.5 0.0
f1 (ppm)

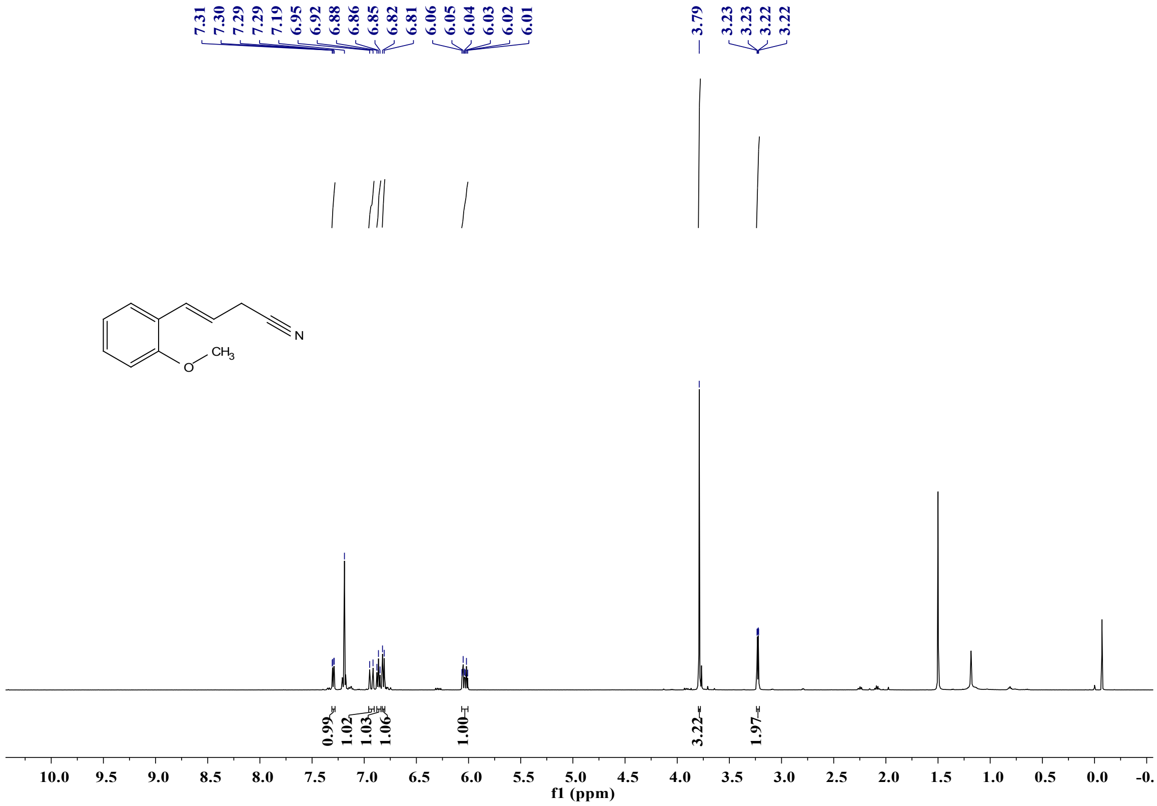
7.31
7.30
7.29
7.29
7.19
6.95
6.92
6.88
6.86
6.85
6.82
6.81
6.06
6.05
6.04
6.03
6.02
6.01
3.79
3.23
3.23
3.22
3.22
N
O
CH3
0.99
1.02
1.03
1.06
1.00
3.22
1.97
10.0 9.5 9.0 8.5 8.0 7.5 7.0 6.5 6.0 5.5 5.0 4.5 4.0 3.5 3.0 2.5 2.0 1.5 1.0 0.5 0.0 -0.
f1 (ppm)

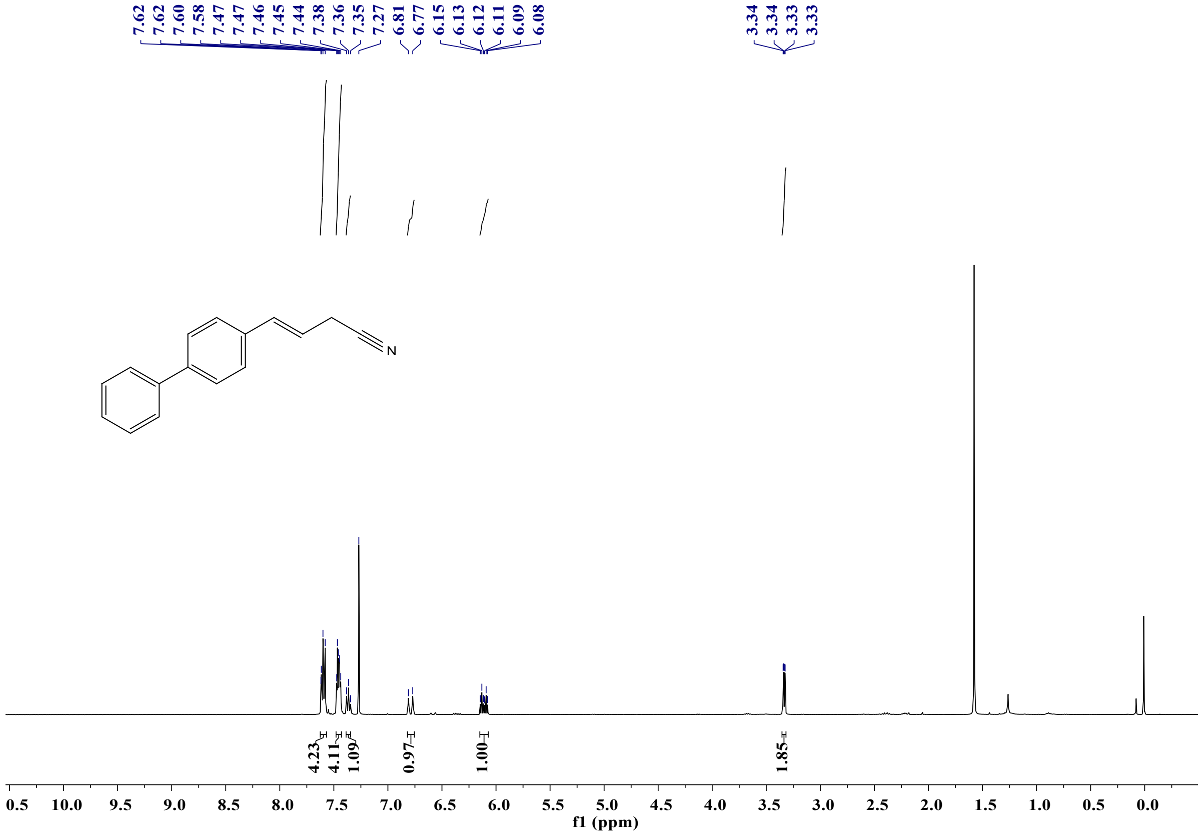
7.62
7.62
7.60
7.58
7.47
7.47
7.46
7.45
7.44
7.38
7.36
7.35
7.27
6.81
6.77
6.15
6.13
6.12
6.11
6.09
6.08
3.34
3.34
3.33
3.33
N
4.23
4.11
1.09
0.97
1.00
1.85
f1 (ppm)

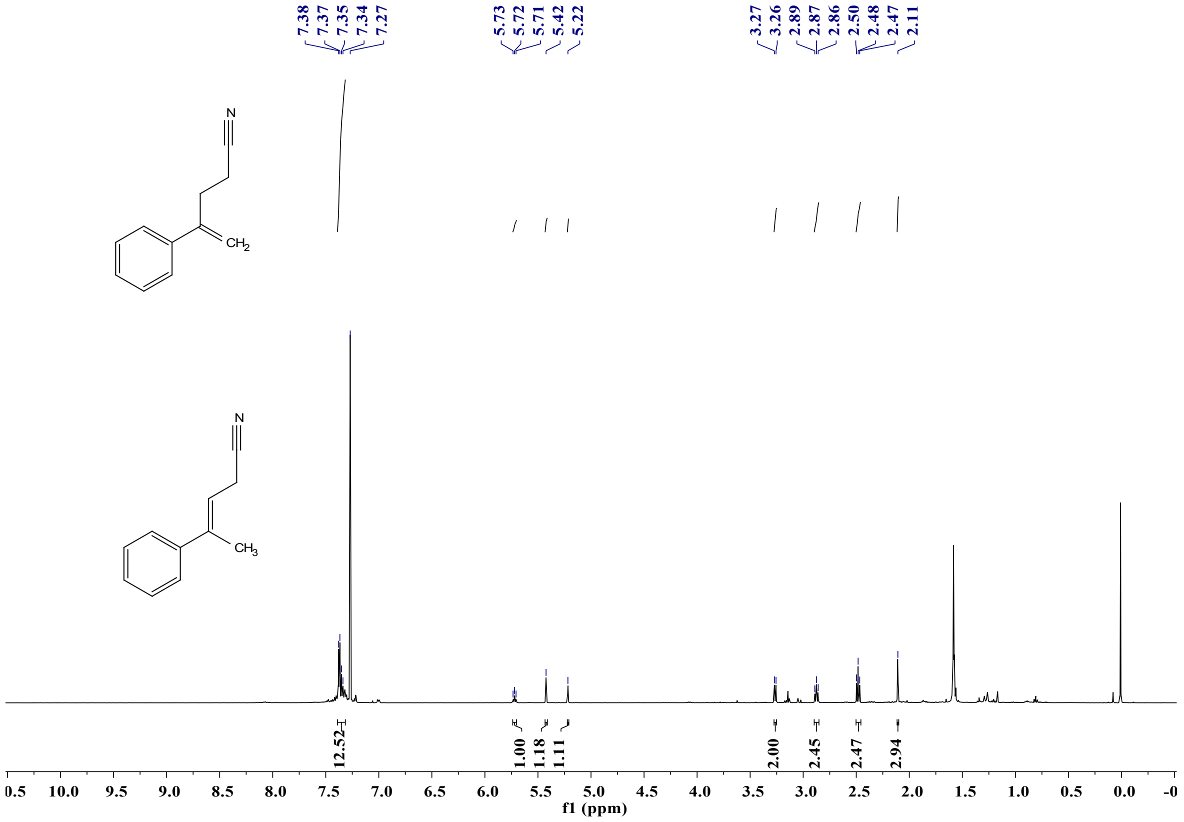
7.38
7.37
7.35
7.34
7.27
5.73
5.72
5.71
5.42
5.22
3.27
3.26
2.89
2.87
2.86
2.50
2.48
2.47
2.11
N
CH2
N
CH3
12.52
1.00
1.18
1.11
2.00
2.45
2.47
2.94
f1 (ppm)

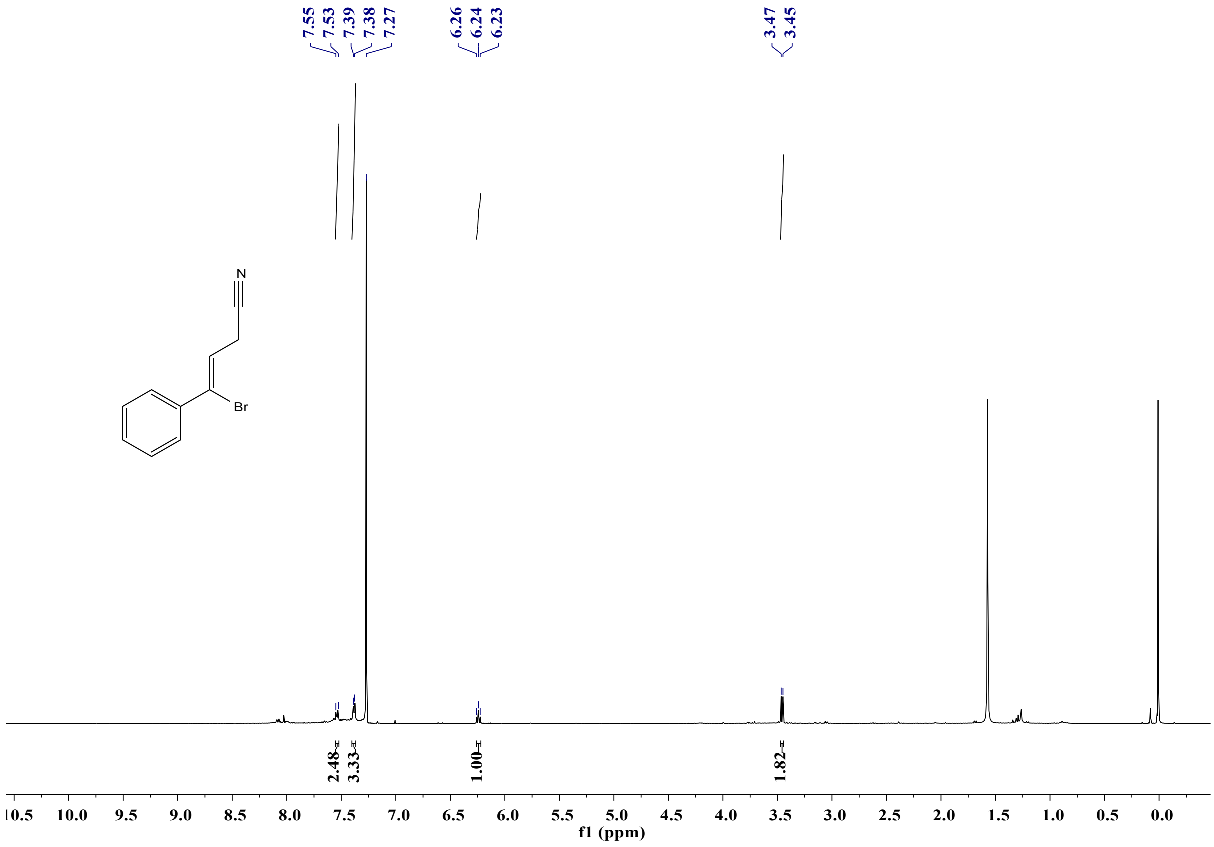
7.55
7.53
7.39
7.38
7.27
6.26
6.24
6.23
3.47
3.45
N
Br
2.48
3.33
1.00
1.82
10.5 10.0 9.5 9.0 8.5 8.0 7.5 7.0 6.5 6.0 5.5 5.0 4.5 4.0 3.5 3.0 2.5 2.0 1.5 1.0 0.5 0.0
f1 (ppm)

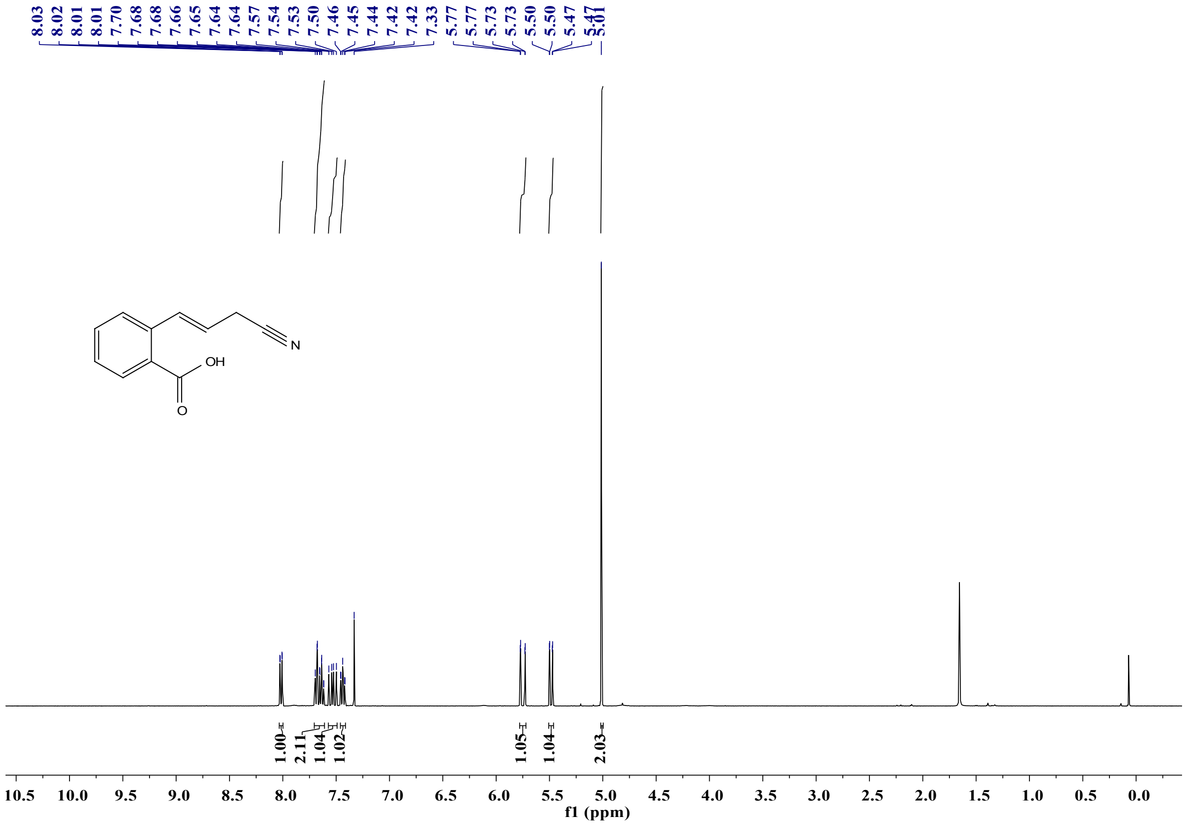
8.03
8.02
8.01
8.01
7.70
7.68
7.68
7.66
7.65
7.64
7.64
7.57
7.54
7.53
7.50
7.46
7.45
7.44
7.42
7.42
7.33
5.77
5.77
5.73
5.73
5.50
5.50
5.47
5.47
5.01
N
OH
O
1.00
2.11
1.04
1.02
1.05
1.04
2.03
10.5 10.0 9.5 9.0 8.5 8.0 7.5 7.0 6.5 6.0 5.5 5.0 4.5 4.0 3.5 3.0 2.5 2.0 1.5 1.0 0.5 0.0
f1 (ppm)

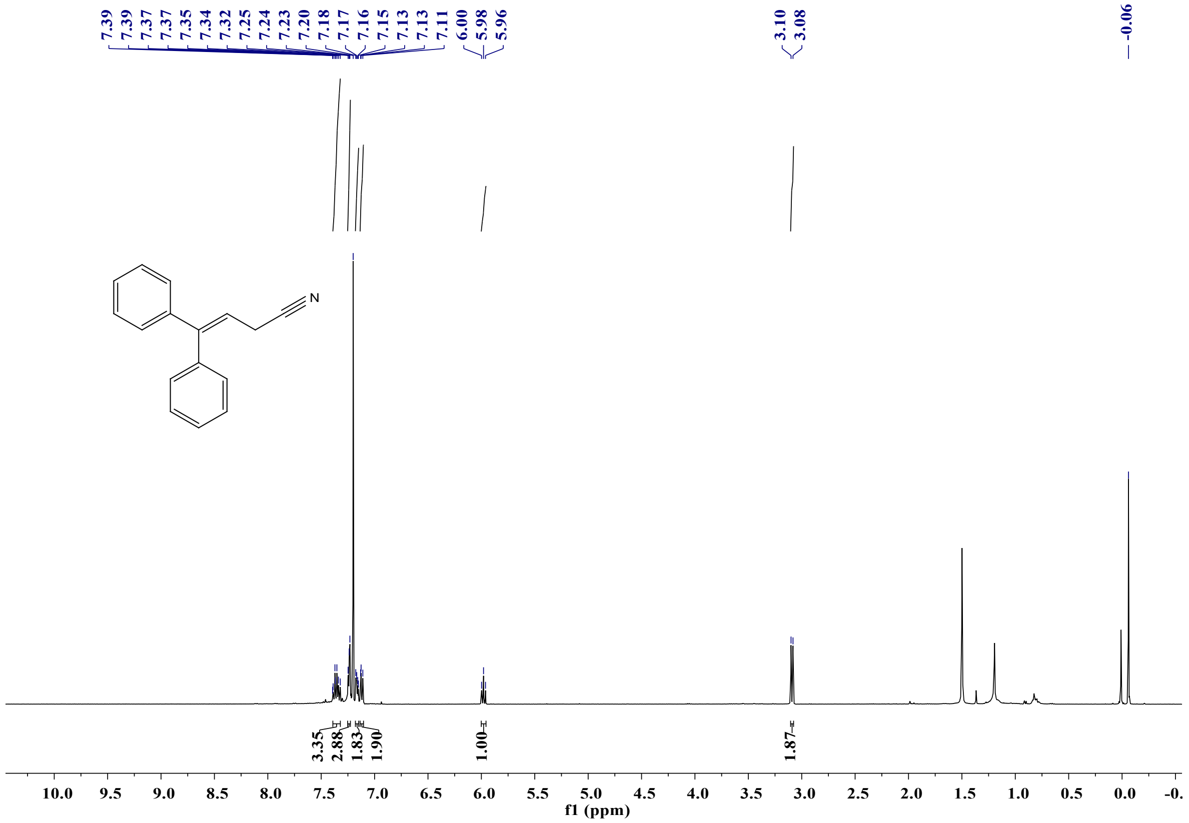
7.39 7.39 7.37 7.37 7.35 7.34 7.32 7.25 7.24 7.23 7.20 7.18 7.17 7.16 7.15 7.13 7.13 7.11 6.00 5.98 5.96
3.10 3.08
-0.06
N
3.35 2.88 1.83 1.90
1.00
1.87
10.0 9.5 9.0 8.5 8.0 7.5 7.0 6.5 6.0 5.5 5.0 4.5 4.0 3.5 3.0 2.5 2.0 1.5 1.0 0.5 0.0
f1 (ppm)

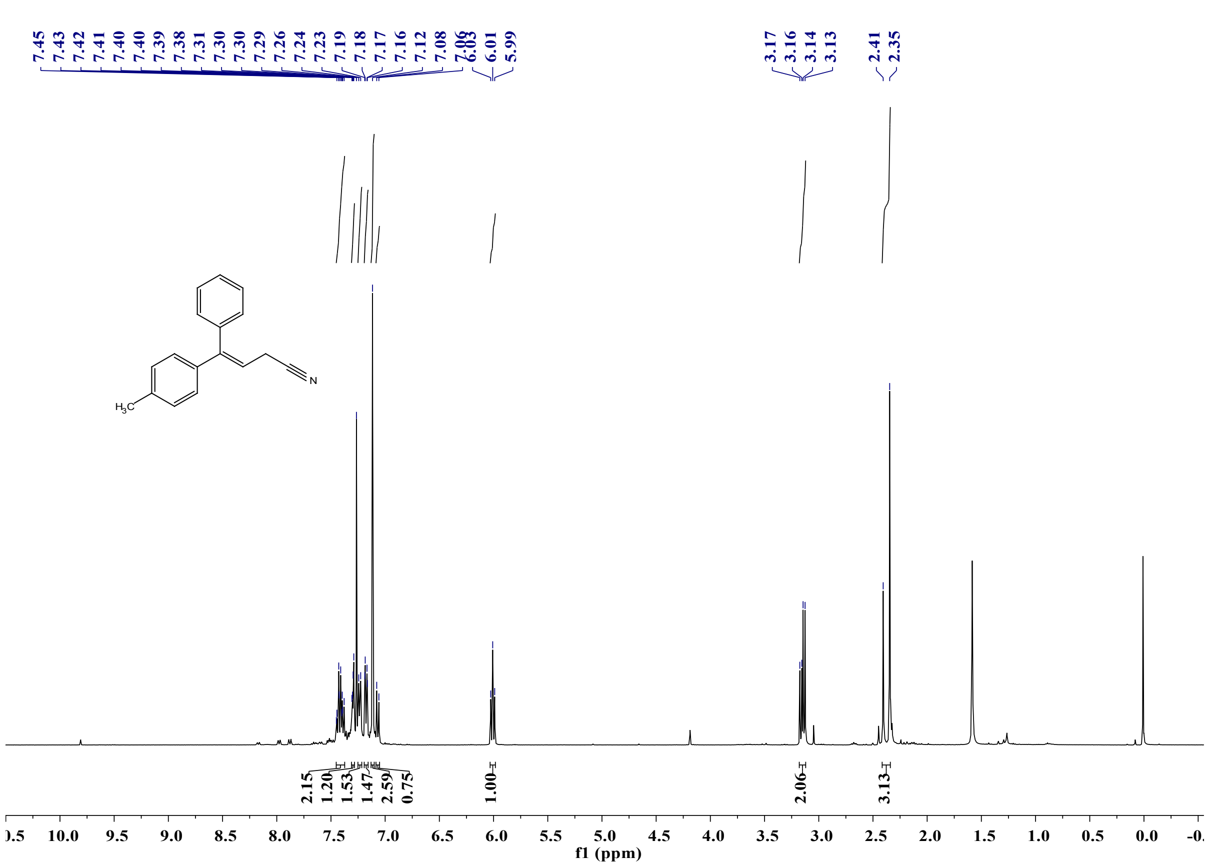
7.45 7.43 7.42 7.41 7.40 7.40 7.39 7.38 7.31 7.30 7.30 7.29 7.26 7.24 7.23 7.19 7.18 7.17 7.16 7.12 7.08 7.06 6.03 6.01 5.99
3.17 3.16 3.14 3.13
2.41 2.35
N
H3C
2.15 1.20 1.53 1.47 2.59 0.75
1.00
2.06
3.13
10.0 9.5 9.0 8.5 8.0 7.5 7.0 6.5 6.0 5.5 5.0 4.5 4.0 3.5 3.0 2.5 2.0 1.5 1.0 0.5 0.0
f1 (ppm)

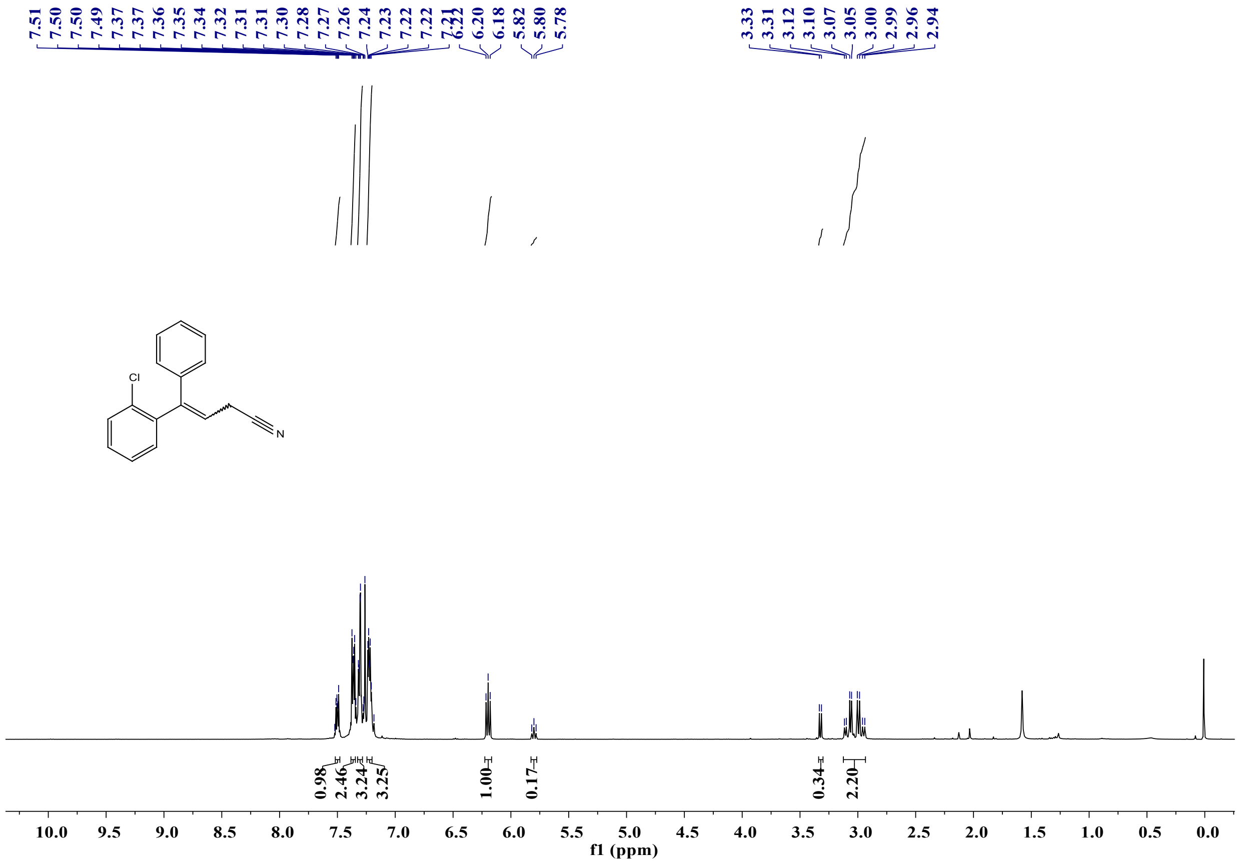
7.51 7.50 7.50 7.49 7.37 7.37 7.36 7.35 7.34 7.32 7.31 7.31 7.30 7.28 7.27 7.26 7.24 7.23 7.22 7.22 7.21 6.22 6.20 6.18 5.82 5.80 5.78
3.33 3.31 3.12 3.10 3.07 3.05 3.00 2.99 2.96 2.94
Cl
N
0.98 2.46 3.24 3.25 1.00 0.17 0.34 2.20
10.0 9.5 9.0 8.5 8.0 7.5 7.0 6.5 6.0 5.5 5.0 4.5 4.0 3.5 3.0 2.5 2.0 1.5 1.0 0.5 0.0
f1 (ppm)

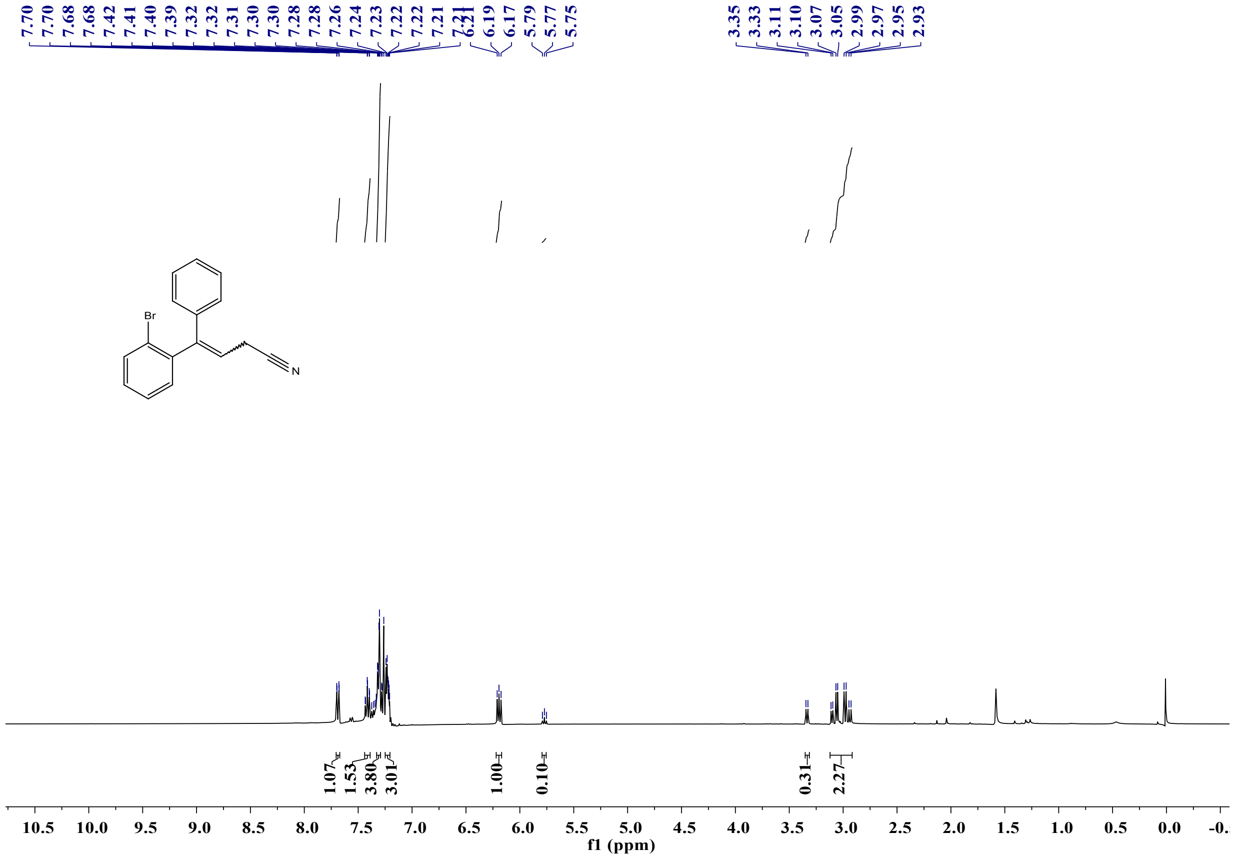
7.70 7.70 7.68 7.68 7.42 7.41 7.40 7.39 7.32 7.32 7.31 7.30 7.30 7.28 7.28 7.26 7.24 7.23 7.22 7.22 7.21 7.21 6.21 6.19 6.17 5.79 5.77 5.75
3.35 3.33 3.11 3.10 3.07 3.05 2.99 2.97 2.95 2.93
Br
N
1.07 1.53 3.80 3.01 1.00 0.10 0.31 2.27
10.5 10.0 9.5 9.0 8.5 8.0 7.5 7.0 6.5 6.0 5.5 5.0 4.5 4.0 3.5 3.0 2.5 2.0 1.5 1.0 0.5 0.0 -0.
f1 (ppm)

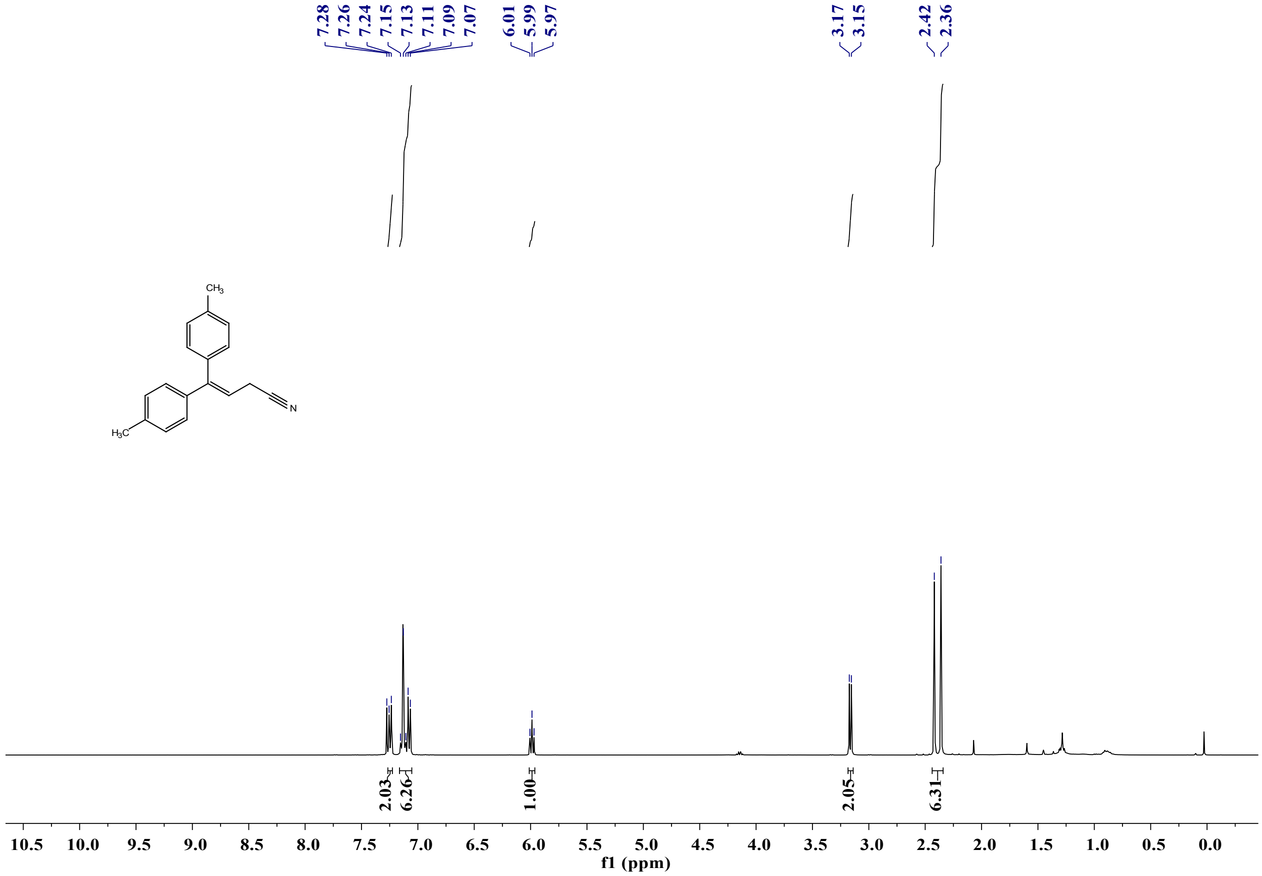
7.28 7.26 7.24 7.15 7.13 7.11 7.09 7.07
6.01 5.99 5.97
3.17 3.15
2.42 2.36
CH3
H3C
N
2.03 6.26 1.00 2.05 6.31
10.5 10.0 9.5 9.0 8.5 8.0 7.5 7.0 6.5 6.0 5.5 5.0 4.5 4.0 3.5 3.0 2.5 2.0 1.5 1.0 0.5 0.0
f1 (ppm)

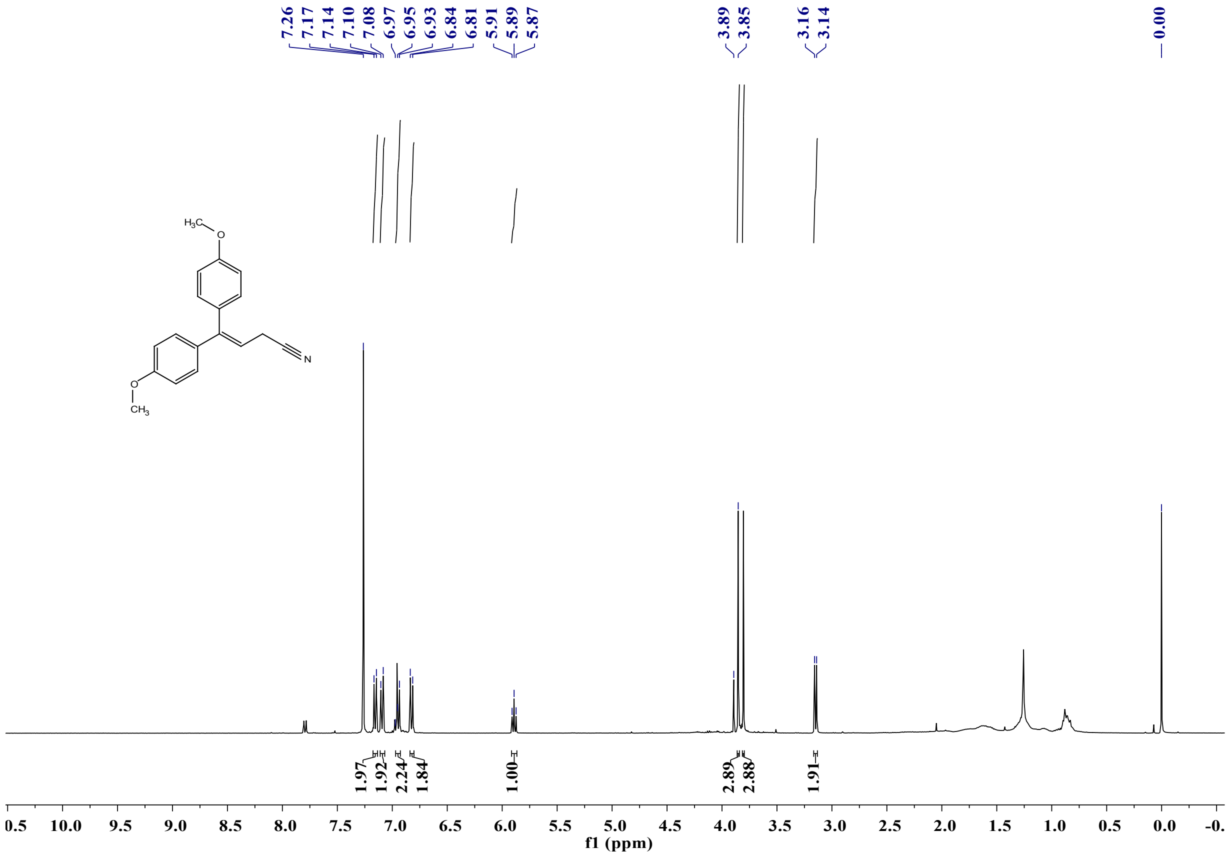
7.26 7.17 7.14 7.10 7.08 6.97 6.95 6.93 6.84 6.81 5.91 5.89 5.87
3.89 3.85
3.16 3.14
0.00
H3C
O
N
CH3
1.97 1.92 2.24 1.84 1.00 2.89 2.88 1.91
0.5 10.0 9.5 9.0 8.5 8.0 7.5 7.0 6.5 6.0 5.5 5.0 4.5 4.0 3.5 3.0 2.5 2.0 1.5 1.0 0.5 0.0 -0.
f1 (ppm)

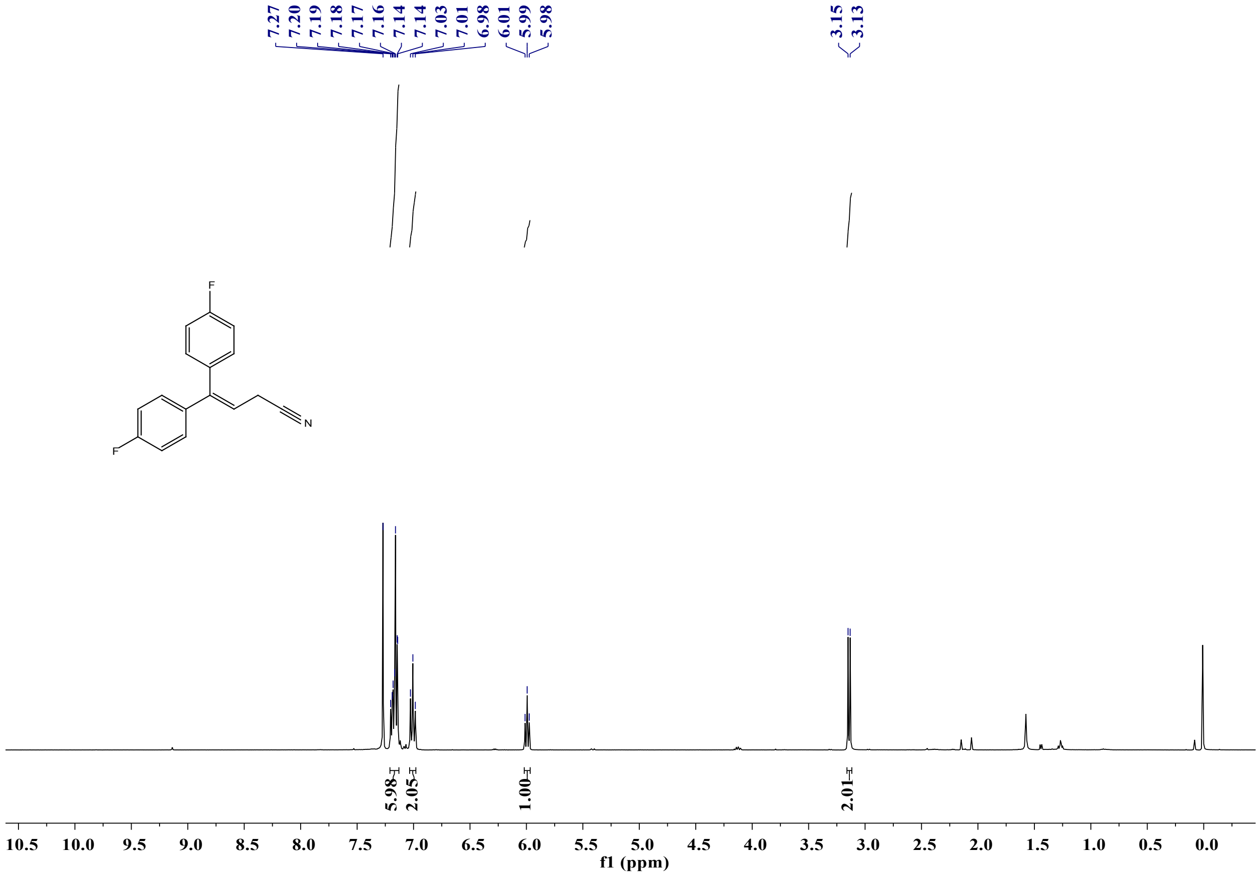
7.27
7.20
7.19
7.18
7.17
7.16
7.14
7.14
7.03
7.01
6.98
6.01
5.99
5.98
3.15
3.13
F
F
N
5.98
2.05
1.00
2.01
10.5 10.0 9.5 9.0 8.5 8.0 7.5 7.0 6.5 6.0 5.5 5.0 4.5 4.0 3.5 3.0 2.5 2.0 1.5 1.0 0.5 0.0
f1 (ppm)

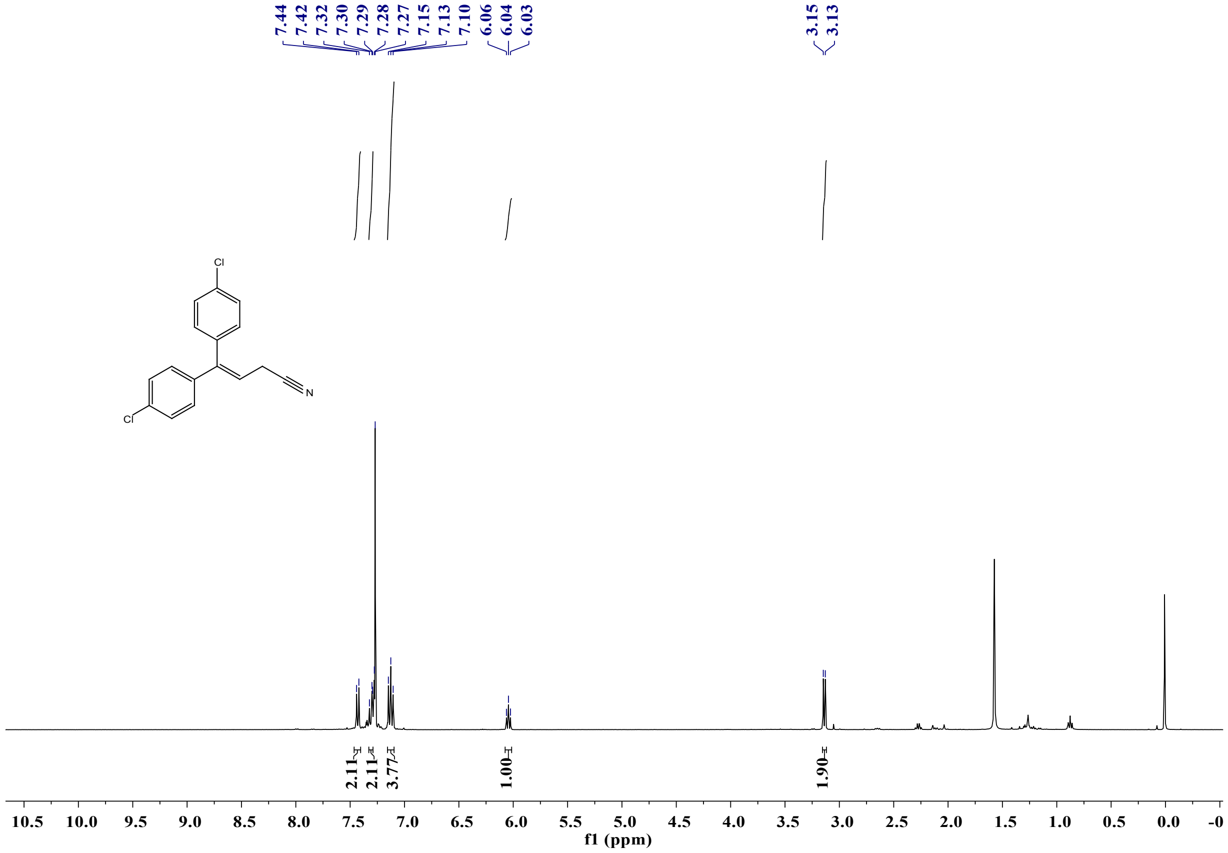
7.44
7.42
7.32
7.30
7.29
7.28
7.27
7.15
7.13
7.10
6.06
6.04
6.03
3.15
3.13
Cl
Cl
N
2.11
2.11
3.77
1.00
1.90
10.5 10.0 9.5 9.0 8.5 8.0 7.5 7.0 6.5 6.0 5.5 5.0 4.5 4.0 3.5 3.0 2.5 2.0 1.5 1.0 0.5 0.0 -0
f1 (ppm)

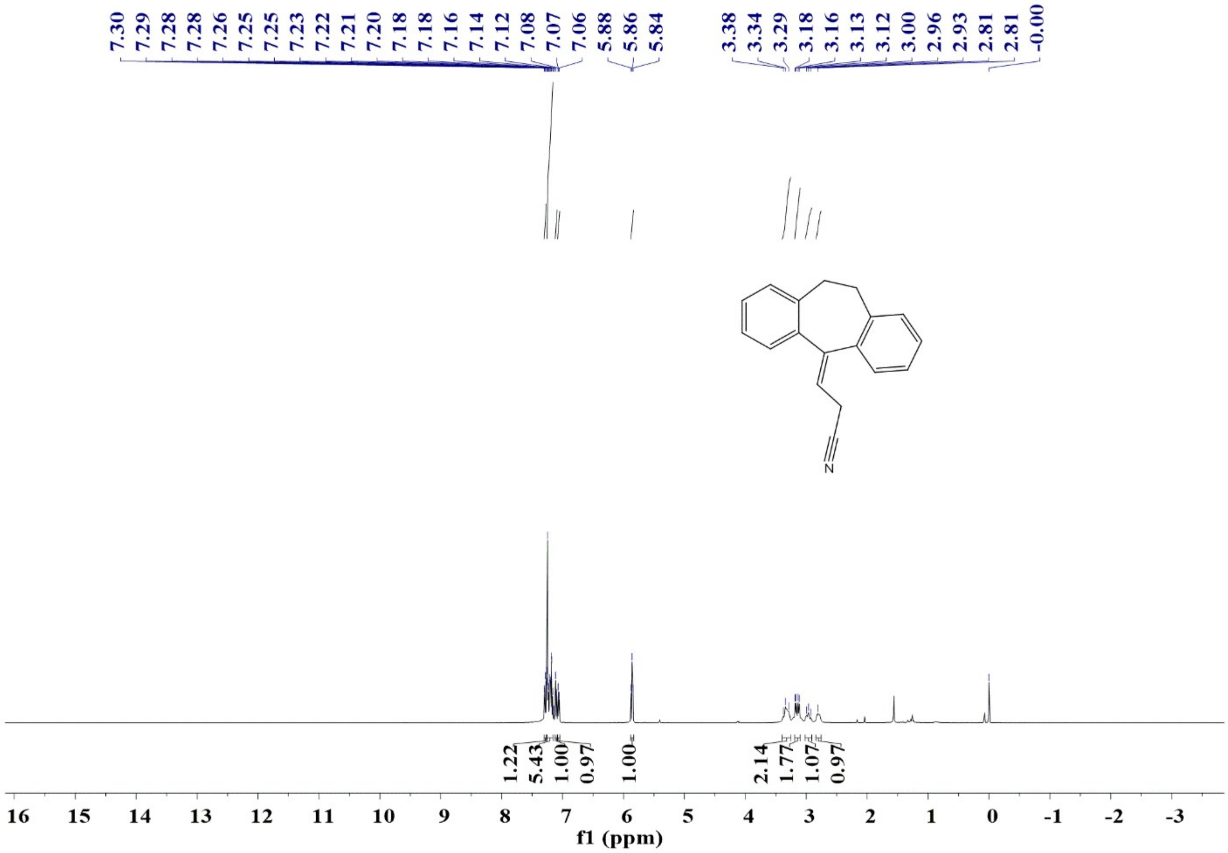

7.30 7.29 7.28 7.28 7.26 7.25 7.25 7.23 7.22 7.21 7.20 7.18 7.18 7.16 7.14 7.12 7.08 7.07 7.06 5.88 5.86 5.84
3.38 3.34 3.29 3.18 3.16 3.13 3.12 3.00 2.96 2.93 2.81 2.81 -0.00
N
1.22 5.43 1.00 0.97 1.00 2.14 1.77 1.07 0.97
16 15 14 13 12 11 10 9 8 7 6 5 4 3 2 1 0 -1 -2 -3
f1 (ppm)

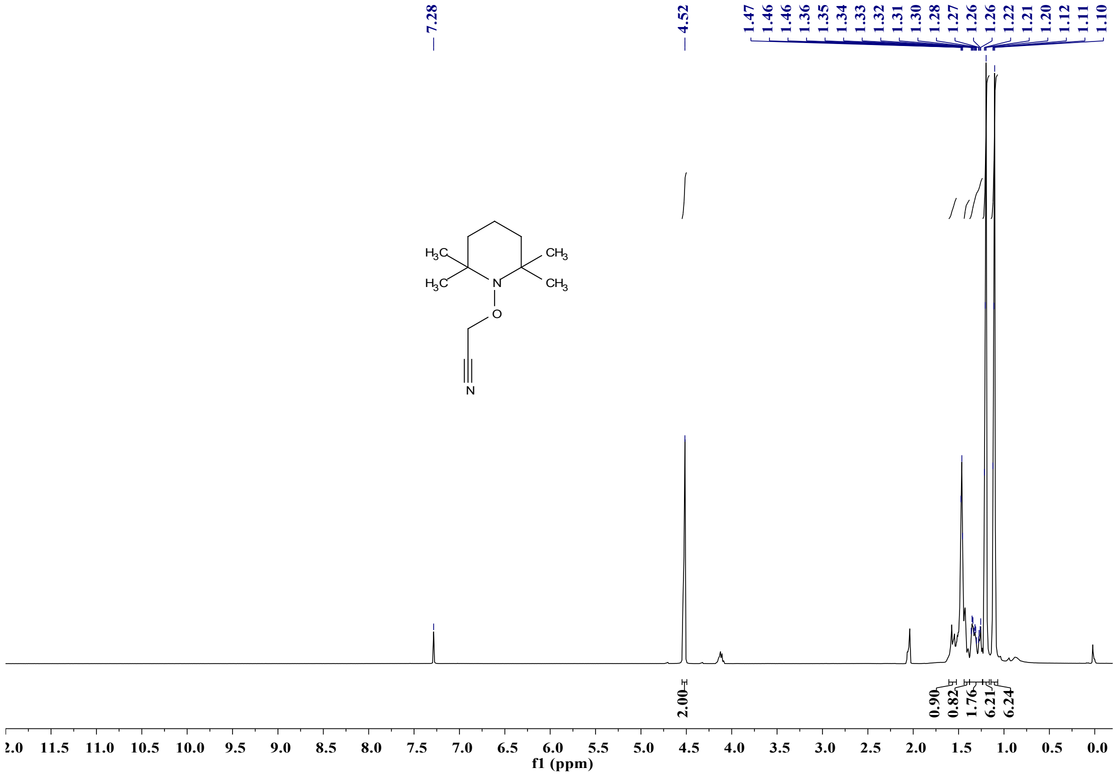

7.28
4.52
1.47 1.46 1.36 1.35 1.34 1.33 1.32 1.31 1.30 1.28 1.27 1.26 1.26 1.22 1.21 1.20 1.12 1.11 1.10
H_3C
CH_3
N
O
2.00
0.90 0.82 1.76 6.21 6.24
11.5 11.0 10.5 10.0 9.5 9.0 8.5 8.0 7.5 7.0 6.5 6.0 5.5 5.0 4.5 4.0 3.5 3.0 2.5 2.0 1.5 1.0 0.5 0.0
f1 (ppm)

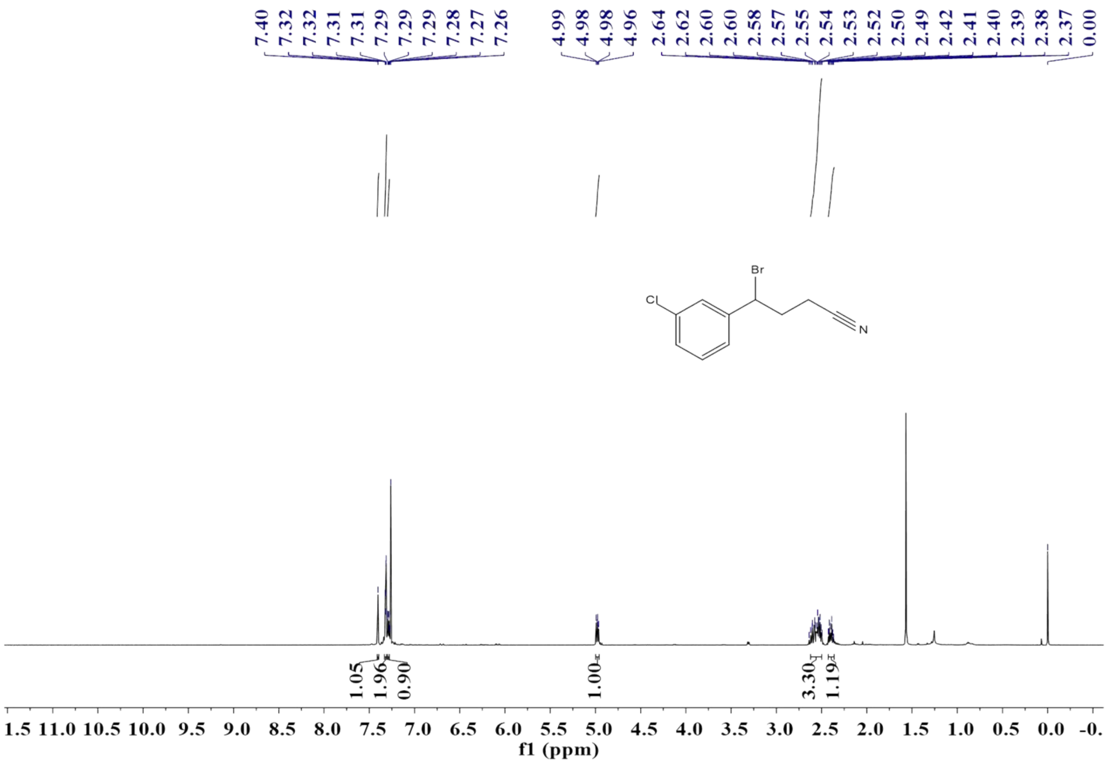

7.40
7.32
7.32
7.31
7.31
7.29
7.29
7.29
7.28
7.27
7.26
4.99
4.98
4.98
4.96
2.64
2.62
2.60
2.60
2.58
2.57
2.55
2.54
2.53
2.52
2.50
2.49
2.42
2.41
2.40
2.39
2.38
2.37
0.00
Br
Cl
N
1.05
1.96
0.90
1.00
3.30
1.19
1.5 11.0 10.5 10.0 9.5 9.0 8.5 8.0 7.5 7.0 6.5 6.0 5.5 5.0 4.5 4.0 3.5 3.0 2.5 2.0 1.5 1.0 0.5 0.0 -0.
f1 (ppm)
